# Interaction of Periplasmic Fab Production and Intracellular Redox Balance in *Escherichia coli* Affects Product Yield

**DOI:** 10.1021/acssynbio.1c00502

**Published:** 2022-01-18

**Authors:** Sophie Vazulka, Matteo Schiavinato, Martin Wagenknecht, Monika Cserjan-Puschmann, Gerald Striedner

**Affiliations:** † Christian Doppler Laboratory for Production of Next-Level Biopharmaceuticals in E. Coli, Department of Biotechnology, Institute of Bioprocess Science and Engineering, University of Natural Resources and Life Sciences, Vienna, Muthgasse 18, 1190 Vienna, Austria; ‡ Department of Biotechnology, Institute of Computational Biology, University of Natural Resources and Life Sciences, Vienna, Muthgasse 18, 1190 Vienna, Austria; § Boehringer Ingelheim RCV GmbH & Co KG, Dr.-Boehringer-Gasse 5-11, 1120 Vienna, Austria

**Keywords:** E. coli, Fab, oxidative folding, periplasmic expression, reactive oxygen species, recombinant protein production, ubiquinone

## Abstract

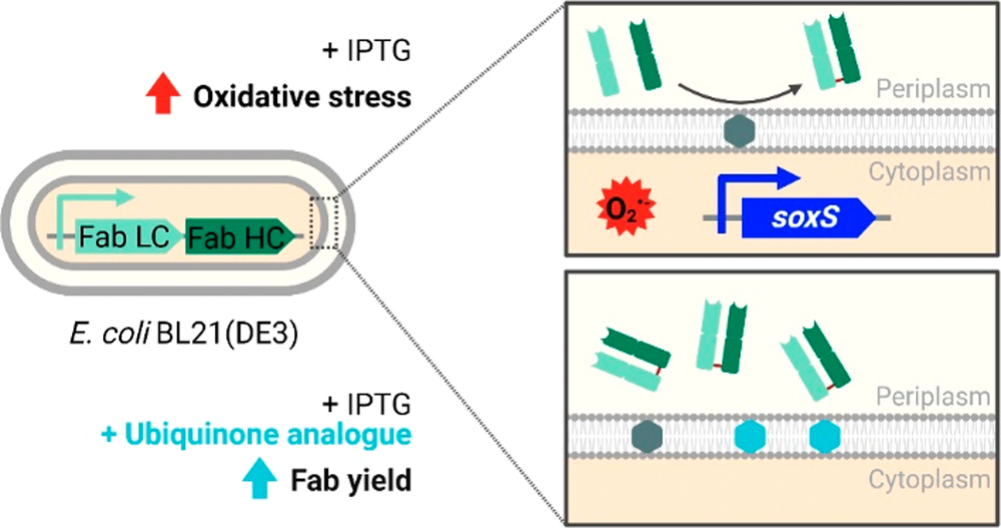

Antibody fragments such as Fab’s require the formation of disulfide bonds to achieve a proper folding state. During their recombinant, periplasmic expression in *Escherichia coli*, oxidative folding is mediated by the DsbA/DsbB system in concert with ubiquinone. Thereby, overexpression of Fab’s is linked to the respiratory chain, which is not only immensely important for the cell’s energy household but also known as a major source of reactive oxygen species. However, the effects of an increased oxidative folding demand and the consequently required electron flux via ubiquinone on the host cell have not been characterized so far. Here, we show that Fab expression in *E. coli* BL21(DE3) interfered with the intracellular redox balance, thereby negatively impacting host cell performance. Production of four different model Fab’s in lab-scale fed-batch cultivations led to increased oxygen consumption rates and strong cell lysis. An RNA sequencing analysis revealed transcription activation of the oxidative stress-responsive *soxS* gene in the Fab-producing strains. We attributed this to the accumulation of intracellular superoxide, which was measured using flow cytometry. An exogenously supplemented ubiquinone analogue improved Fab yields up to 82%, indicating that partitioning of the quinone pool between aerobic respiration and oxidative folding limited ubiquinone availability and hence disulfide bond formation capacity. Combined, our results provide a more in-depth understanding of the profound effects that periplasmic Fab expression and in particular disulfide bond formation has on the host cell. Thereby, we show new possibilities to elaborate cell engineering and process strategies for improved host cell fitness and process outcome.

Monoclonal antibodies and antibody-derived molecules are extensively used for various applications including therapeutics and diagnostics. With an increase in global annual sales from $84 billion to $163 billion between 2014 and 2019,^1^ they represent the fastest growing segment of the biopharmaceutical market.^2^ Due to cost-effective cultivation, microbial expression systems such as *Escherichia coli* represent a utile alternative for smaller-sized antibody fragments that do not rely on glycosylation for functionality.^3^ One format that retains its antigen-binding capacity (Fab) consists of the light chain (LC) and two domains of the heavy chain (HC) of an immunoglobulin G (IgG) molecule. Each chain is composed of a constant (C_L_, C_H_1) and a variable domain (V_L_, V_H_). Antigen binding is mediated by the complementary determining regions within the variable domains of the Fab. For correct folding, five disulfide bonds are required.^4^

Different approaches to express Fab’s in *E. coli* have been described. Efforts have been made to enable production in the cytoplasm, whereby investigated strategies aimed at expression as inclusion bodies (IBs) and subsequent refolding or at manipulating the prevalent reducing conditions, thereby allowing the formation of disulfide bonds.^5,6^ However, the method commonly used is fusion of the Fab’s HC and LC to a signal sequence for translocation across the inner membrane (IM) into the periplasmic space which is the only bacterial compartment that naturally provides the oxidizing conditions to enable the formation of disulfide bonds.^7^

In bacterial systems, various factors such as intracellular degradation or aggregation, and toxicity effects of the recombinant protein frequently lead to low product yields.^8^ In eukaryotic expression systems, oxidative stress has also been associated with the production of heterologous proteins. These studies implicate involvement of disulfide bond formation and breakage, secretion, and endoplasmic reticulum (ER) stress in eliciting the stress response.^9−12^ Bacteria lack a compartment equivalent to the ER. Instead, oxidative folding is mediated by the thiol:disulfide oxidoreductase DsbA and the thiol:quinone oxidoreductase DsbB within the periplasm. DsbA donates its disulfide bond to a nascent protein and gets re-oxidized by the IM protein DsbB in order to remain catalytic. DsbB in turn is regenerated by transferring the electrons to ubiquinone (UQ_8_). Oxidative folding in the bacterial periplasm is therefore directly linked to the respiratory chain.^13,14^

The respiratory chain of *E. coli* is extremely versatile, enabling the cell to optimize its energy household under various conditions. Multiple dehydrogenases and terminal oxidases for the utilization of different electron donors and acceptors are linked by the quinone pool. Combination of isozymes leads to different degrees of coupling between electron and proton transport.^15^ The resulting proton motive force (PMF) is fueling ATP formation through oxidative phosphorylation. During aerobic conditions, NADH is oxidized by NADH dehydrogenases I (NDH I, *nuo* operon) and II (NDH II, *ndh*), enabling varying flux distribution between them. NDH I recovers energy from NADH oxidation (2 H^+^/e^–^), while NDH II is noncoupling.^16^ Electron flow is directed mainly via UQ_8_ and to a lesser extent via menaquinone (MQ) and its precursor demethylmenaquinone (DMQ).^17,18^ The terminal oxidases cytochrome bo, bd I, and bd II transfer the electrons from the quinone pool to O_2_. At high O_2_ levels, mainly cytochrome bo is utilized.^19,20^

Partially reduced oxygen species are generated as inevitable byproducts of aerobic metabolism when O_2_ is reduced in single-electron reactions. The resulting products superoxide (O_2_^•–^), hydrogen peroxide (H_2_O_2_), and hydroxyl radical (OH^•^) generally referred to as reactive oxygen species (ROS) are therefore ubiquitous.^21−23^ Protective mechanisms to keep ROS at harmless levels have evolved in aerobes. Superoxide dismutases (SODs) and catalases/peroxidases prevent accumulation of endogenous O_2_^•–^ and H_2_O_2_, respectively.^24^ Mutant strains deprived of the protective enzymes are poisoned by increased levels of O_2_^•–^ and H_2_O_2_ when cultivated in the presence of O_2_.^25−27^ Basic defense mechanisms are quickly overcome when cells experience sudden elevated levels of ROS. The resulting imbalance of formation and elimination of ROS causes oxidative stress. Damage to proteins (through oxidation of flavin cofactors, metal centers, and amino acids), DNA, and phospholipids is the consequence. *E. coli* has a second, inducible line of defense to deal with oxidative stress. Diverse cellular antioxidant mechanisms are executed by redox stress sensors SoxR and OxyR.^21,22,28^ The two transcription factors are activated by oxidation of [2Fe–2S] clusters^29,30^ and cysteine residues,^31,32^ respectively. OxyR responds to H_2_O_2_;^30^ however, the signals sensed by SoxR are still a matter of debate.^33^ A known trigger of SoxR activation is accumulation of O_2_^•–^ and nitric oxide.^34−37^ Recent research has also indicated other mechanisms of direct metal center oxidation and interference with SoxR inactivation (reduction) pathways as alternative activators.^23,25,38^ Oxidized SoxR in turn activates transcription of *soxS*, a gene coding for a secondary transcription factor. Targets of the SoxRS and OxyR regulons scavenge ROS, boost synthesis of reducing equivalents, repair oxidatively damaged proteins and DNA, and help to provide redox-resistant isozymes for sensitive enzymes.^21−23,38,39^

We hypothesized that overexpression of Fab’s in the periplasmic space requires higher oxidative folding activity to provide the disulfide bonds necessary for folding and consequently increased flux of electrons via UQ_8_ and through the respiratory chain. Since the respiratory chain is a known source of ROS,^40^ this might lead to disturbances of the redox balance and to metabolic changes. To address these interdependencies as a possible consequence of periplasmic Fab expression, we conducted lab-scale, fed-batch cultivations of a set of recombinant *E. coli* BL21(DE3) strains expressing four different Fab’s fused to the post-translational translocation signal sequence of the OmpA protein of *E. coli* (ompA^SS^). We used genome-integrated expression systems to avoid plasmid-mediated metabolic load and other confounding factors as described elsewhere.^41^ Strong T7-based systems with a single Fab gene copy were chosen, in order to achieve a sufficiently strong cell response to Fab production, while reducing the metabolic load.^42^

In this study, we present evidence that perturbation of the host cell’s redox balance is indeed a consequence of expressing Fab’s in the periplasm of *E. coli* BL21(DE3) and that this interaction can be utilized for the improvement of production. We monitored increased oxygen consumption rates (qO_2_) and cell lysis as a consequence of Fab production in fed-batch cultivations. In fed-batch-like microtiter cultivations, we detected higher levels of intracellular O_2_^•–^ in Fab-producing strains. Supplementing a UQ_8_ analogue to the growth medium led to increased Fab yields, indicating UQ_8_ deficiency during Fab expression. RNA sequencing (RNA-seq) revealed elevated transcript levels of the O_2_^•–^-inducible *soxS* gene at later stages of the fed-batch fermentations as well as changes in the gene expression behavior of NADH dehydrogenases.

## Results and Discussion

For correct folding, Fab molecules require one inter- and four intrachain disulfide bonds. Twenty years ago, Bader et al. showed that oxidative folding in the periplasmic space is directly linked to the respiratory chain via the quinone pool.^13^ The main goal of this study was to investigate the interplay between increased oxidative folding demand in the periplasm, concomitant electron flux through the respiratory chain via UQ_8_, and effects thereof on host strain and process performance.

### Increased Oxygen Consumption Rates of Fab-Producing Strains in Glucose-Limited Fed-Batch Cultivations

**Figure 1:**
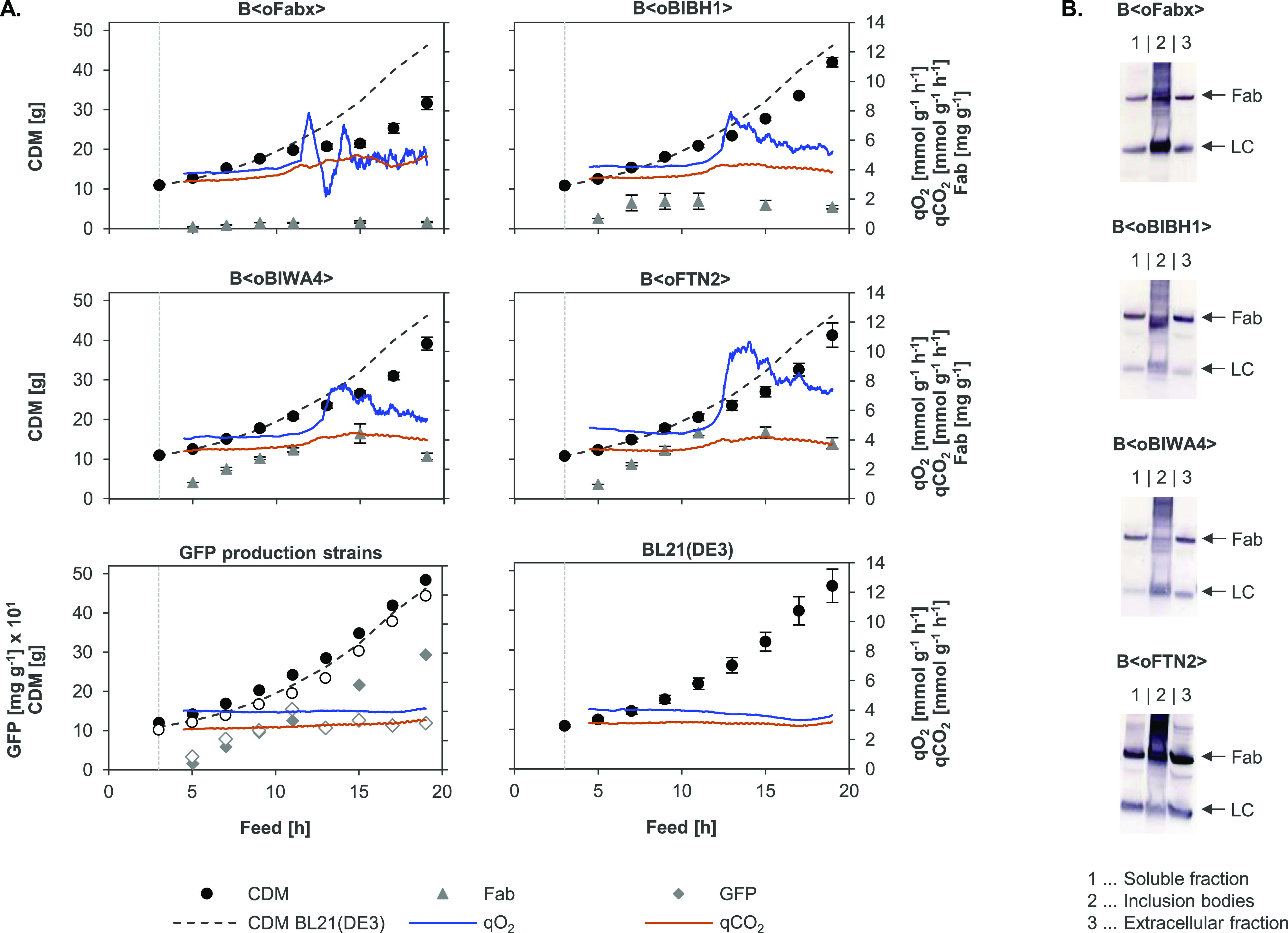
(A) CDM [g], total (intra- and extracellular) soluble Fab yields [mg g^–1^ CDM] and qO_2_ and qCO_2_ [mmol g^–1^ CDM h^–1^] during glucose-limited fed-batch cultivations. GFPmut3.1 and sfGFP yields were plotted in [mg g^–1^ CDM] × 10^1^. Cultivations of the wildtype reference BL21(DE3) and the Fab-producing strains B⟨oFabx⟩, B⟨oBIBH1⟩, B⟨oBIWA4⟩, and B⟨oFTN2⟩ were performed in triplicate (mean + SEM, *n* = 3). Both GFP-producing strains were cultivated once. CDM and yield of cytoplasmic GFPmut3.1 of B⟨GFPmut3.1⟩ are shown by filled circles (●) and diamonds (gray ⧫), respectively, and CDM and yield of periplasmic sfGFP of B⟨dsfGFP⟩ are shown by empty circles (○) and diamonds (◊), respectively. Online data for qO2 and qCO_2_ are presented as moving average with a period of 60 data points (equals approx. 1 min) for a single representative experiment. For the GFP production strains, qO_2_ and qCO_2_ are only shown for B⟨GFPmut3.1⟩ since the measurements were very similar to B⟨dsfGFP⟩. Induction is indicated by the vertical dashed, gray line. The CDM of the BL21(DE3) wildtype strain is shown in the graphs of all recombinant strains for comparison. (B) Fab expression patterns of endpoint samples (after 16 h induction) analyzed by LC-specific WB. (1) Soluble and (2) IB fractions and (3) recombinant protein found extracellularly in the culture supernatant are shown. Fractions loaded in each lane were adjusted to the same biomass for comparability. Bands corresponding to Fab (approx. 50 kDa) and LC (approx. 25 kDa) are indicated by arrows.

To observe the effects of periplasmic Fab expression on the host cells under relevant production conditions, we conducted lab-scale fed-batch cultivations of Fab-producing strains (strain abbreviations are listed in Table 1). Biomass accumulation and total specific soluble Fab titers including the intra- and extracellular fractions are shown in Figure 1A. The wildtype strain BL21(DE3) and BL21(DE3) expressing green fluorescent protein (GFP) as “easy-to-produce” protein^43^ without disulfide bonds were included as reference systems. Two variants of GFP were used: cytosolic GFPmut3.1 and periplasmic superfolder GFP (sfGFP) that were translocated by fusion to the signal sequence of the DsbA protein (dsbA^SS^). All Fab-producing strains showed reduced accumulation of biomass compared to the wildtype strain, which reached a final biomass of 46.19 g of cell dry mass (CDM). Impact on growth was most pronounced in B⟨oFabx⟩ with 31.63 g of CDM, followed by B⟨oBIWA4⟩ with 39.12 g, B⟨oFTN2⟩ with 41.27 g, and B⟨oBIBH1⟩ with 42.00 g of final CDM. Cytosolic GFPmut3.1 production had no negative impact on cell growth and resulted in a final CDM of 48.39 g for B⟨GFPmut3.1⟩. Periplasmic expression of sfGFP led to slightly reduced biomass of 44.36 g for B⟨dsfGFP⟩. Intra- and extracellular Fab titers were analyzed from cell lysates and culture supernatant, respectively, using enzyme-linked immunosorbent assay (ELISA). GFP was quantified fluorometrically. Total specific Fab titer at the end of the production phase was the highest for FTN2 with 3.76 mg g^–1^ CDM, followed by BIWA4 with 2.88 mg g^–1^ CDM and BIBH1 with 1.46 mg g^–1^ CDM. The lowest titer was obtained for Fabx with 0.44 mg g^–1^ CDM. In addition to Fab molecules, also considerable amounts of unassembled LCs were detected. Different ratios of Fab to unassembled LCs in the soluble and IB fraction of cell lysates at the end of the production process are shown in LC-specific western blots (WBs) in Figure 1B. Unassembled HCs were hardly detectable in HC-specific WBs, presumably due to proteolysis. GFP was expressed at substantially higher levels compared to Fab’s. Cytosolic GFPmut3.1 reached a concentration of 293.67 mg g^–1^ CDM, while periplasmic expression levels of sfGFP were lower at 119.10 mg g^–1^ CDM.

**Table 1:** Used *E. coli* Strains and Genome-Integrated Expression Systems

strains and abbreviations	description	source
*E. coli* BL21(DE3)	F-ompT gal dcm lon hsdSB(rB–mB−) λ(DE3 [lacI lacUV5-T7p07 ind1 sam7 nin5])	NEB
B⟨oFabx⟩	BL21(DE3) expressing ompA^SS^-Fabx	(41)
B⟨oBIBH1⟩	BL21(DE3) expressing ompA^SS^-BIBH1	(41)
B⟨oBIWA4⟩	BL21(DE3) expressing ompA^SS^-BIWA4	(41)
B⟨oFTN2⟩	BL21(DE3) expressing ompA^SS^-FTN2	(41)
B⟨GFPmut3.1⟩	BL21(DE3) expressing GFPmut3.1	(92)
B⟨dsfGFP⟩	BL21(DE3) expressing dsbA^SS^-sfGFP	in house

Upon comparison of the online data measured during the different cultivations, it became obvious that Fab expression led to an increase in the qO_2_ compared to the BL21(DE3) wildtype and BL21(DE3) expressing either of the two GFP variants. The reference strains showed a constant qO_2_ of approx. 4 mmol g^–1^ h^–1^ throughout the process as expected^44^ (Figure 1A). The value is in accordance with numbers reported for glucose-limited growth at a rate of μ = 0.1 h^–1^.^45^ The strains expressing the four different Fab fragments exhibited a rather constant qO_2_ of approx. 4 mmol g^–1^ h^–1^ at the beginning of the process. However, concomitant with a deviation of the biomass from wildtype growth, the qO_2_ sharply increased up to approx. 8 mmol g^–1^ h^–1^ for B⟨oFabx⟩, B⟨oBIBH1⟩, and B⟨oBIWA4⟩ and approx. 10 mmol g^–1^ h^–1^ for B⟨oFTN2⟩. After reaching a peak, the qO_2_ slowly dropped again. The CO_2_ formation rate (qCO_2_) stayed rather constant for all strains. The surge in qO_2_ was accompanied by increasing levels of extracellular product (Figures 1B and S1) due to cell lysis (confirmed by measurement of increasing DNA levels in the culture supernatant using Hoechst dye; data not shown). Therefore, lower CDM yields of Fab-producing strains could also partly be attributed to loss of biomass by lysis.

There are multiple possible influence factors that could be responsible for the observed increase in qO_2_. Expression of Fab’s and the formation of disulfide bonds needed to reach their correct conformation lead to an increased oxidative folding demand, which in turn would require increased flux of electrons via UQ_8_. The respiratory chain is known as the major contributor to the formation of O_2_^•–^,^40^ which is scavenged by SODs.^24^ Single-electron transfer reactions to O_2_ are a prerequisite for the formation of O_2_^•–^; hence, increased O_2_^•–^formation would require higher O_2_ consumption. Assuming that all disulfide bonds are formed correctly and do not require breakage, re-formation leads to a theoretical consumption of 1 O_2_/LC and HC (4 e^–^/LC and HC) and 2.5 O_2_/Fab molecule (10 e^–^/Fab). Since considerable IB formation, production of unassembled LCs, and the formation of incorrect Fab derivatives as described by Schimek et al.^46^ were observed, it was not possible to quantify total recombinant protein production and calculate the respective amount of O_2_ needed as an electron acceptor. However, even at high recombinant protein titers, disulfide bond formation alone could not account for 100% (Fabx, BIBH1, and BIWA4) to 150% (FTN2) increase in qO_2_ when assuming stoichiometric O_2_ consumption. It has been discovered that the formation of disulfide bonds in secretory proteins is connected to the formation of ROS in eukaryotes.^10,47^ ER-resident proteins Ero1p and protein disulfide isomerases catalyze the formation of disulfides analogous to bacterial DsbB and DsbA.^48^ Ero1p regenerates by directly transferring electrons to O_2_ in a flavin-dependent reaction, thereby producing one molecule of H_2_O_2_ per disulfide bond.^49^ However, nonstoichiometric amounts of ROS produced by oxidative folding of overexpressed proteins have been determined experimentally.^9,48^ Incorrect disulfide bonds are broken and need to be reformed to reach their native state. Repeated breakage and re-formation of non-native disulfide bonds resulting in futile cycles have been proposed as a possible explanation for increased qO_2_ and ROS formation (e.g., when an uneven number of cysteines are present or folding is slow).^9^ In *E. coli,* disulfide bond isomerization is carried out by oxidoreductase DsbC in concert with the IM protein DsbD. Electrons are donated by the NADPH pool and transferred to DsbD by cytoplasmic thioredoxin.^50^ High levels of non-native disulfide bonds possibly lead to elevated O_2_ demand when they have to be re-formed. However, proteins with consecutive disulfide bonds such as Fab’s generally do not rely on DsbC and *dsbC* deletion has indeed been reported not to affect human Fab activity or yield when produced in *E. coli*.^51^ Additionally, supplementation of 10 mM glutathione which is described to aid reshuffling of disulfides and thereby improve titers of recombinant, disulfide bond-containing proteins^52^ led to decreased instead of increased Fab yields in both fed-batch-like microtiter and lab-scale fed-batch cultivations in our hands (data not shown). The effect on cell growth was not consistent and therefore not conclusive. Since the preliminary experiments did not show the anticipated improvements (as described by Kumar et al.^53^), we focused on other strategies to improve Fab production, even though the underlying mechanisms would be worth further investigation.

Campani et al. described that the metabolic burden exerted by recombinant protein production can impact qO_2_.^54^ However, in our case, constant qO_2_ during cultivation of the GFP-producing strains demonstrated that high-level expression of a recombinant protein and consequently increased ATP demand alone was not sufficient to increase qO_2_. Therefore, qO_2_ depended solely on the growth rate and the nature of the used carbon source in both GFP-producing strains. Furthermore, for periplasmic expression SecA-mediated, ATP- and PMF-driven translocation of the recombinant proteins across the IM is necessary.^55^ In contrast to cytosolic GFPmut3.1, expression of sfGFP and Fab’s required translocation and hence additional ATP, which could have influenced qO_2_. Nevertheless, expression of periplasmic sfGFP did not cause an increase in qO_2_; hence, energy consumption by translocation did not seem to have an impact on qO_2_.

Since rather high levels of cell lysis were occurring, starting at approx. 11 h of feed with up to 66% of the product found extracellularly at the end of the process (Figure S1), cellular components in the culture broth might also have influenced qO_2_. Cells are able to utilize nutrients liberated by lysed cells, which leads to higher qO_2_ as observed during the death phase and cryptic growth in the stationary phase.^56^

Finally, metabolic shifts and changes in respiration have been described upon perturbation of the respiratory chain and the PMF, which could be connected to increased oxidative folding activity. Manipulation of respiration has even been utilized for engineering metabolite distribution.^57−60^ Castan et al. also found increased levels of mixed acid fermentation metabolites upon use of O_2_-enriched process air.^61^

It is unclear to what extent each of the possibilities mentioned above impacted the observed increase in qO_2_. Probably, the surge and subsequent decline of qO_2_ were impacted by a combination of changes in metabolism, cell lysis, and oxidative folding. In any case, the need to use pure O_2_ in the in-gas stream to maintain dissolved oxygen (DO) at 30% demonstrated the pronounced effects of Fab production on the host cells. It needs to be mentioned that especially, cell lysis influenced total process performance, since it was associated with not only higher amounts of the extracellular product but also genomic DNA found in the fermentation broth. Loss of product and possibly product quality and decreased processability in downstream processing through higher viscosity due to extracellular DNA would be problematic consequences and need to be considered during process design.

### Accumulation of Intracellular Superoxide in Fab-Producing Strains

Even though the qO_2_ increase observed in Fab-producing strains during fed-batch cultivations was presumably not directly caused by disulfide bond formation, we assumed a connection to oxidative folding. This prompted us to test if Fab expression was indeed connected to the formation of ROS, more specifically O_2_^•–^. CellROX Green reagent was used to determine intracellular O_2_^•–^ formation. This weakly fluorescent dye enters the cell and, when oxidized, becomes strongly fluorescent and binds to double-stranded DNA. According to the manufacturer, the dye is sensitive to oxidation by O_2_^•–^ and OH^•^, but not H_2_O_2_, ONOO^–^, NO, and ClO^–^. It has also been demonstrated by McBee et al. that H_2_O_2_ treatment did not cause fluorescence increase in CellROX Green-stained *E. coli* cells.^62^ Fab-producing strains B⟨oFabx⟩, B⟨oBIBH1⟩, B⟨oBIWA4⟩, and B⟨oFTN2⟩ and the BL21(DE3) wildtype reference strain were grown in fed-batch-like cultivations in the microtiter format to achieve a higher amount of parallelization for including controls. CellROX Green-stained cells were analyzed flow cytometrically, and induced cultures were compared to noninduced ones 12 h after induction of Fab production. Cultures of induced BL21(DE3) wildtype were used as a reference. Wildtype BL21(DE3) treated with the redox cycling drug menadione (MD) that causes an increase in the CellROX Green signal due to the formation of O_2_^•–^,^62^ served as a positive control.

**Figure 2:**
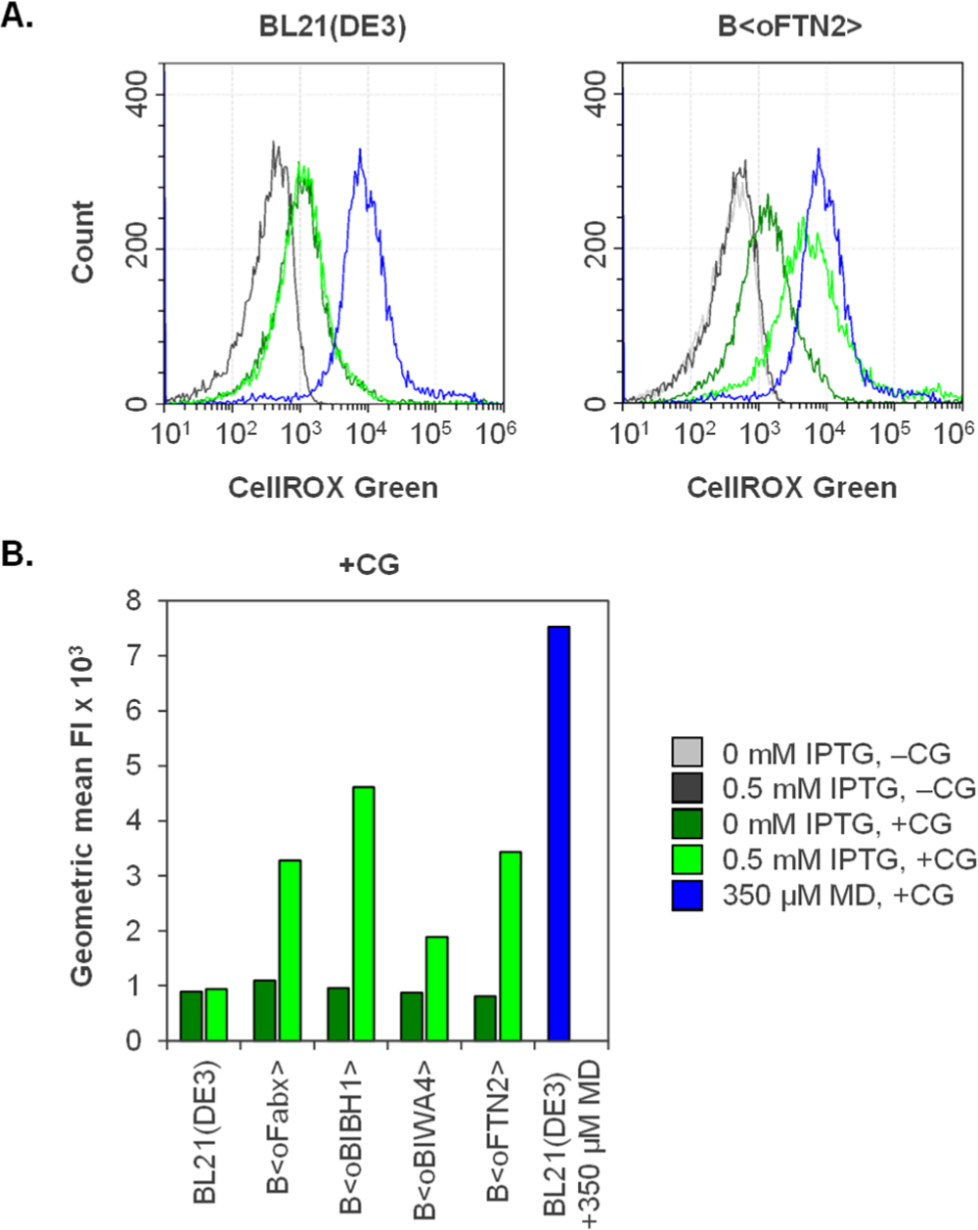
Flow cytometric analysis of Fab-producing strains and the BL21(DE3) wildtype strain grown in fed-batch-like cultivations in the microtiter format after 12 h of cultivation/induction. Fluorescence at 488/525 nm of noninduced (0 mM IPTG) and induced cells (0.5 mM IPTG) was analyzed without (−CG) and with (+CG) staining with CellROX Green reagent. Wildtype BL21(DE3) treated with 350 μM MD served as a positive control. (A) Single representative measurements of BL21(DE3) and B⟨oFTN2⟩ are shown in histogram plots. The positive control in blue is shown in both diagrams. (B) GeoMeanFI × 10^3^ of noninduced and induced samples with CellROX Green staining. All samples were analyzed in biological triplicate (*n* = 3, variance ⟨ 18%).

Fab expression patterns of the cultivations are shown in LC-specific WBs of the soluble and IB fractions in Figure S2. Histograms of the cell count plotted against the fluorescence intensity (FI) of BL21(DE3) and B⟨oFTN2⟩ are shown in Figure 2A as examples. B⟨oFabx⟩, B⟨oBIBH1⟩, and B⟨oBIWA4⟩ are shown in Figure S3A. Noninduced [0 mM β-d-1-thiogalactopyranoside (IPTG)] and induced cultures (0.5 mM IPTG) were measured with (+CG) and without (−CG) CellROX Green staining to detect changes in autofluorescence and avoid introduction of artifacts. Of the tested Fab-producing strains, only B⟨oFabx⟩ showed a slight increase in autofluorescence upon induction. Increased forward (FSC) and side scatter (SSC) signals indicated that altered autofluorescence was probably caused by changes in cell size and morphology (Figure S3B). For all strains, an increase in the SSC signal accompanied by a fluorescence shift could be seen for noninduced cultures upon staining with CellROX Green (Figure S3C). The geometric mean of the FI (GeoMean FI) was obtained for all samples. GeoMean FI values of the samples without CellROX Green staining were subtracted from stained cultures for every strain, for noninduced and induced cultures separately. The resulting values are plotted in Figure 2B. The CellROX Green-stained BL21(DE3) wildtype reference exhibited unchanged GeoMean FI regardless of IPTG addition. Fab-producing strains without the addition of IPTG exhibited GeoMean FI comparable to that of the wildtype reference. However, the induced, CellROX Green-stained Fab-producing strains all showed substantially higher GeoMean FI compared to the noninduced samples. Induction caused the strongest GeoMean FI increase in B⟨oBIBH1⟩, followed by B⟨oFabx⟩ and B⟨oFTN2⟩. Induced B⟨oBIWA4⟩ showed lower, but still clearly elevated GeoMean FI in the induced cultures compared to the noninduced ones. As expected, the addition of 350 μM MD to the BL21(DE3) wildtype led to an FI shift of nearly one log step in the positive control (Figure 2A) which equals an almost eightfold increase in the GeoMean FI (Figure 2B).

Hereby, we clearly demonstrated that induction of Fab expression in *E. coli* BL21(DE3) production strains caused increased oxidation of an O_2_^•–^-sensitive dye. The observed effects were somewhat lower than for the MD-treated positive control, indicating less-pronounced O_2_^•–^ formation elicited by Fab production under the present conditions than by the action of the redox cycling drug. The intracellular site of O_2_^•–^ formation in our experiment remains unclear. However, the respiratory chain has generally been identified as the major source of O_2_^•–^ (but not H_2_O_2_) in the cell.^40^ It is tempting to speculate that at least a part of the detected O_2_^•–^ was formed by an increasing number of single-electron transfer reactions to O_2_ within the respiratory chain, directly or indirectly caused by oxidative folding of the recombinant protein. One possibility is a higher rate of O_2_^•–^ formation by the terminal oxidases simply due to higher flux of electrons from oxidative folding. Another possible site of O_2_^•–^ formation is NDH II.^18^ NDH II is known to produce O_2_^•–^ via autoxidation of its flavin cofactor^21,40^ and there are two explanations for increased autoxidation. Higher flux through NDH II instead of NDH I or a lack of downstream electron acceptors causes electrons to remain on the autoxidizable flavin.^63^ Electrons backed up on NDH II were identified as responsible for O_2_^•–^ formation in membrane vesicles obtained from a UQ_8_-deficient mutant.^64^ Hence, partitioning of the UQ_8_ pool between NADH oxidation and DsbB regeneration during oxidative folding of the recombinant proteins might lead to a limitation of available UQ_8_ and in further consequence increased O_2_^•–^ formation. Another explanation for higher O_2_^•–^ formation, also assuming UQ_8_ deficiency, is the transfer of electrons to MQ. DsbB can use MQ as an alternative electron acceptor under aerobic and anaerobic conditions.^13^ Higher production of O_2_^•–^ in *ubiAC* mutants that lack UQ_8_ and lower levels of O_2_^•–^ in *menA* mutants with a deletion in the MQ synthesis pathway have been observed.^18^ The two quinones have different redox potentials (+0.113 V for UQ_8_ and −0.074 V for MQ), which is why MQ could also transfer electrons directly to O_2_ and thereby contribute to O_2_^•–^ formation.

### Exogenous Supplementation of Coenzyme Q_1_ (CoQ_1_) to Increase Fab Yields

Insufficient amounts of oxidized UQ_8_ within the cell might lead to increased formation of O_2_^•–^ in the respiratory chain and to an insufficient oxidative folding capacity. Therefore, we tested if supplementation of the artificial UQ_8_ analogue CoQ_1_ to the growth medium could increase the yield of correctly folded, soluble Fab. CoQ_1_ has been used in other studies to control respiration in a *ubiAC* mutant.^65^ Compared to endogenous UQ_8_, the polyprenyl hydrophobic tail contains less isoprenyl units (1 instead of 8 in UQ_8_).^66^

In a first approach, we tested the impact of supplementing 5 μM CoQ_1_ to an induced shake flask culture of B⟨oFTN2⟩, where the growth rate was not limited by glucose feeding. CoQ_1_ addition led to improved growth with a final OD_600_ of 3.2 compared to 2.0 of the control without CoQ_1_ after 4 h of Fab production. The soluble FTN2 band observed in LC-specific WB analysis was slightly increased when CoQ_1_ was added (Figure S4). Since fed-batch cultivation is more industrially relevant, batch experiments were not pursued further. Nevertheless, the experiment showed that boosting the available UQ_8_ pool seemed to positively impact ubiquinone availability for cell growth and oxidative folding under conditions without C-limitation.

**Figure 3:**
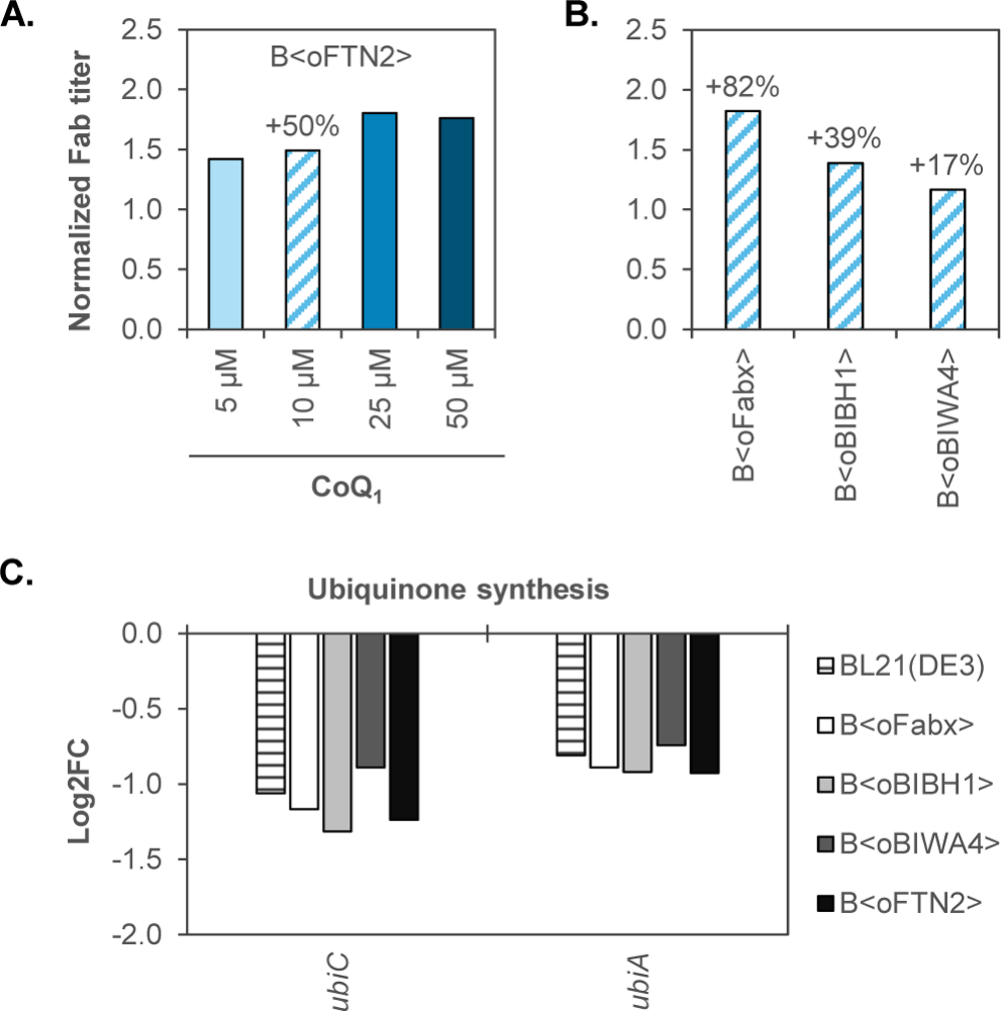
(A) FTN2 produced in fed-batch-like microtiter cultivations with different concentrations of the UQ_8_ analogue CoQ_1_ (0–50 μM). Biological duplicates were analyzed, and volumetric FTN2 yields were normalized to the cultivation without CoQ_1_ addition (*n* = 2). (B) Fab yields obtained in fed-batch-like microtiter cultivations of B⟨oFabx⟩, B⟨oBIBH1⟩, and B⟨oBIWA4⟩ with supplementation of 10 μM CoQ_1_. Volumetric Fab yields were normalized to yields obtained without CoQ_1_ addition for each respective Fab. One cultivation was analyzed for Fabx, BIBH1, and BIWA4 with analytical variance ⟨10%. (C) Log2FC of genes involved in the UQ_8_ synthesis pathway that were differentially expressed (α ≤ 0.05) after 12 h of induction in fed-batch cultivations relative to the sample drawn prior to induction as determined by RNA-seq (*n* = 3).

The effect of supplementing different concentrations of CoQ_1_ was further analyzed using the strain B⟨oFTN2⟩ in fed-batch-like microtiter cultivations. The total UQ_8_ content of aerobically growing cells has been measured at approx. 1090 nmol g^–1^ CDM.^67^ In our setup (a final CDM of maximum 10 g L^–1^ in 800 μL working volume), this equals a concentration of approx. 11 μM CoQ_1_. Therefore, 0 μM, 5 μM (approx. 0.5× the endogenous intracellular UQ_8_ concentration), 10 μM (1×), 25 μM (2.5×), and 50 μM (5×) CoQ_1_ were supplemented to the cultivations at induction in addition to IPTG. Induction of recombinant protein production caused a slight decrease in final biomass compared to the noninduced cultures (9.8 g L^–1^). Induced cultures reached a final biomass of 7.9 g L^–1^ at all tested CoQ_1_ concentrations, including the reference without CoQ_1_ addition. Intracellular Fab yields were analyzed using ELISA. Extracellular amounts of Fab detected in LC-specific WB were negligible (data not shown) and were therefore not considered. A positive effect of all tested CoQ_1_ concentrations on FTN2 yield was observed. Fab yields obtained in cultivations with CoQ_1_ supplementation were normalized to the cultivation without CoQ_1_. An increase in FTN2 yield between 1.4- and 1.8-fold could be achieved (Figure 3A). In a follow-up cultivation, the remaining Fab-producing strains B⟨oFabx⟩, B⟨oBIBH1⟩, and B⟨oBIWA4⟩ were supplemented with 0 or 10 μM CoQ_1_ at induction (Figure 3B). In all tested Fab strains, CoQ_1_ improved Fab production to different degrees. Fabx yield was increased by 82%, BIBH1 by 39%, BIWA4 by 17%, and FTN2 as previously determined, by 50%.

The fact that UQ_8_ plays a vital role in proper functioning of the respiratory chain (and hence energy household and growth)^18,65,68,69^ and oxidative folding^13,70^ has been well-established. A recent study found that disulfide bond formation was impaired in *E. coli* through growth on long-chain fatty acids, which causes increased levels of NADH.^71^ The authors reasoned that increased electron flux through the respiratory chain by NADH oxidation led to UQ_8_ deficiency and showed that providing UQ_8_ exogenously can restore disulfide bond formation. Comparably, increased oxidative folding demand exerted by periplasmic Fab expression seemed to exhaust the cells’ UQ_8_ pool, which could be counteracted by supplementation of CoQ_1_. Another study found that mutant *E. coli* forming only 20% of the normal amount of UQ_8_ showed decreased growth and decreased oxidase activity, even though UQ_8_ was still present in excess with respect to cytochrome bo.^72^ This highlights that sufficient availability of UQ_8_ is crucial to maintain both the respiratory chain and oxidative folding activity intact. By increasing Fab yields substantially upon supplementation of CoQ_1_, we showed that UQ_8_ indeed seemed to be a limiting factor during Fab expression.

### Downregulation of *ubi* Genes during Glucose-Limited Fed-Batch Cultivations

To get a comprehensive view of the host cell response elicited upon Fab production stress on the transcription level, we investigated changes in gene expression over the course of the fed-batch cultivations using RNA-seq. We analyzed differential gene expression (DGE) in samples drawn 2, 12, and 16 h after induction relative to the noninduced sample after 3 h of feed. DGE profiles of the Fab-producing strains were compared to the BL21(DE3) wildtype strain to exclude changes in gene expression dependent on the process conditions or production of T7 RNA polymerase due to IPTG addition.

The UQ_8_ synthesis pathway in *E. coli* comprises multiple genes in different genomic locations (*ubiCA*, *ubiD*, *ubiEJB*, *ubiHI*, *ubiX*, *ubiG*, and *ubiF*).^66^ The genes encoding the enzymes dedicated to the first two steps in UQ_8_ synthesis, *ubiC* (chorismate pyruvate lyase) and *ubiA* (4-hydroxybenzoate octaprenyltransferse), were negatively affected by the process conditions in fed-batch cultivations after 12 h (Figure 3C) and 16 h of induction (Figure S8). Downregulation of the *ubiCA* operon was comparable in Fab-producing strains and the wildtype reference with a log2 fold change (log2FC) between 0.7 and 1.3 (see Table S1). Hence, Fab expression that exhausts the cells’ UQ_8_ pool did not trigger UQ_8_ synthesis. This is in accordance with Kwon et al.,^73^ who reported low expression levels of the operon during growth on fermentable carbon sources (such as glucose used in our study). Increased expression has been observed in cells provided with the oxidizable carbon source glycerol under aerobic conditions.^73,74^

### Transcription Activation of *soxS* and *marRAB* upon Fab Expression

**Figure 4:**
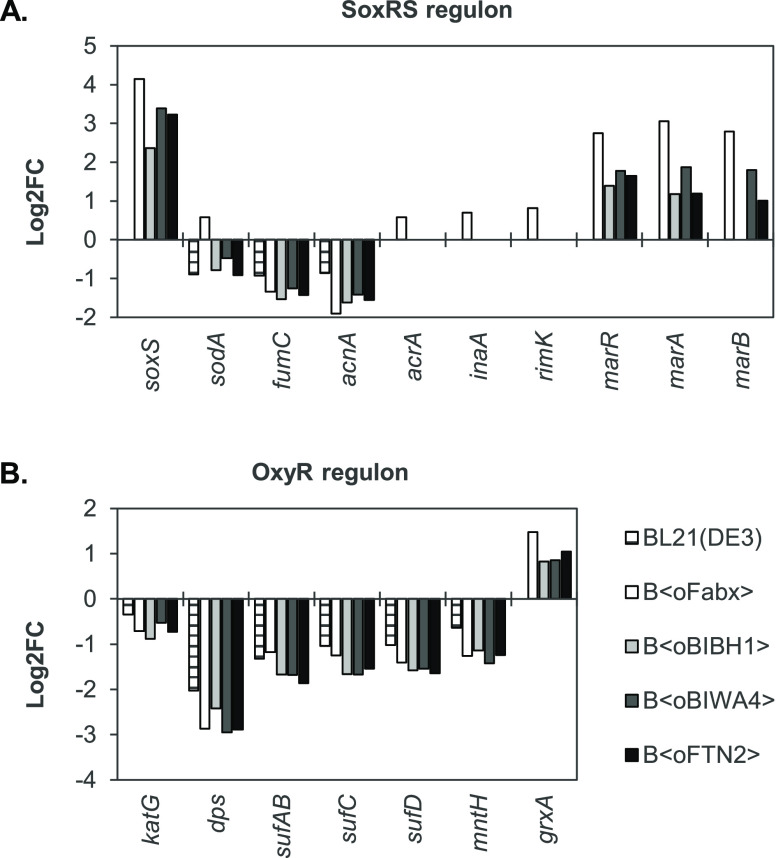
DEGs (α ≤ 0.05) of members of the (A) SoxRS and (B) OxyR regulons as determined by RNA-seq. Log2FC after 12 h of induction relative to the sample drawn prior to induction are shown for the Fab-producing strains and the BL21(DE3) wildtype (*n* = 3).

When ROS such as O_2_^•–^ are formed at a higher rate than the cells’ basal defense mechanisms can disarm them, *E. coli* relies on two known lines of defense against oxidative stress, which are inducible on the transcription level: the SoxRS and the OxyR regulons. Since we observed elevated intracellular O_2_^•–^ levels in Fab-producing strains in microtiter cultivations, we focused on genes that are activated either by SoxR/SoxS or OxyR. Log2FC of members of the SoxRS and OxyR regulons that were differentially expressed after 12 h of induction is shown in Figure 4A,B, respectively.

We observed activation of *soxS* transcription in all Fab-producing strains 12 h after induction. Log2FC varied between 2.4 in B⟨oBIBH1⟩ and 4.2 in B⟨oFabx⟩ (see Table S1). Transcript levels of *soxS* were still elevated after 16 h of production albeit less-pronounced than earlier in the process (Figure S5). The only known activator of *soxS* transcription is SoxR, which triggers *soxS* transcription upon oxidation of its [2Fe2S] cluster.^75,76^ Hence, increased *soxS* transcript levels revealed activation of SoxR. Oxidation of SoxR is mediated, for example, by O_2_^•–^, which we showed accumulates in Fab-producing strains. No *soxS* upregulation was observed after 2 h of production. At the earlier stages of the production phase, the cells’ basal lines of defense apparently were sufficient to keep formed O_2_^•–^at harmless levels. After prolonged Fab production, basal defenses seemed to be overwhelmed, and the cells had to resort to inducible mechanisms to mitigate the formed O_2_^•–^. It was reported by Baez and Shiloach^77^ that the use of O_2_-enriched process air can lead to SoxRS activation in *E. coli*. A contribution thereof cannot be excluded; however, O_2_ levels in the in-gas stream did not necessarily coincide with *soxS* upregulation levels. Although the % O_2_ after 16 h induction was equal or higher compared to 12 h, *soxS* transcript levels decreased between the two time points (Figure S6A). This was confirmed by qPCR measurements of samples drawn from a B⟨oFTN2⟩ cultivation at additional time points. Increased *soxS* levels could be detected not only after 12 but also after 6 h of induction when no pure O_2_ had been added (Figure S6B). Additionally, *soxS* transcript levels were higher after 10 h than 12 h of induction, which does not coincide with the use of higher levels of O_2_, but with ceased productivity toward the end of the process (and parallelly decreasing *soxS* transcription upregulation). Furthermore, the increased levels of O_2_^•–^ in Fab-producing strains cultivated in microtiter plates were independent of factors such as O_2_-enriched process air.

The list of genes activated by the secondary transcription factor SoxS is continuously being extended. Surprisingly, hardly any of the known SoxS target genes (e.g. *zwf*, *nfo*, *fur*, *fldAB*, ...^23,39^) were upregulated in Fab-producing strains, despite activated *soxS* transcription. B⟨oFabx⟩ showed slight upregulation (0.5 ⟨ log2FC ⟨ 0.8) of *sodA*, *acrA*, *inaA*, and *rimK*. Some SoxS-activated genes (*fumC* and *acnA* in all strains, and *sodA* in all strains, except B⟨oFabx⟩) were down- rather than upregulated. This can be attributed to process conditions since the same expression pattern was observed in wildtype BL21(DE3). The multiple-antibiotic-resistance operon *marRAB* was upregulated in all Fab-producing strains but not in the wildtype reference. Overlap between SoxRS and MarRAB operon and activation of *marRAB* transcription by SoxS have been reported and ascribed to the structural similarity between the transcription factors SoxS and MarA.^39,75,78^ Gene *ybjC* was described to be activated by MarA and SoxS^39^ and was also moderately upregulated in B⟨oFabx⟩ in our setup.

OxyR is activated when its cysteine residues are oxidized by H_2_O_2_.^32^ Targets include, for example, *ahpCF*, *fur*, *trxA*, and *gor*, most of which were not differentially expressed in our experiments. The small RNA OxyS was not captured due to size exclusion steps in the library preparation method. Genes *katG*, *dps*, the *sufABCD* operon, and *mntH* were downregulated in Fab-producing strains and the BL21(DE3) wildtype alike. Interestingly, all Fab-producing strains showed upregulated transcript levels of *grxA* (0.8 > log2FC > 1.5) as the sole OxyR target in response to Fab production.

RNA-seq provides a snapshot of global transcription but no information about protein abundances. SoxS expression is regulated not only on transcription but also on the translation level by the small RNA MgrR in an Hfq-binding manner^79^ and by proteolysis.^80^ Therefore, no clear answer can be derived from the transcriptome data, why almost no target genes of the SoxRS regulon were upregulated, while *soxS* transcription was activated. Possible explanations could be interferences with other regulatory pathways that affect expression either of SoxS or its target genes. For example, expression of MnSOD (*sodA* gene product) is regulated by four global transcription regulators in addition to SoxS (Fur, AcrA, Fnr, and IHF)^81^ and in a post-translational fashion.^82^

OxyR is activated by concentrations of H_2_O_2_ beyond 0.1 μM. However, high activity of peroxidase prevents accumulation of endogenously formed H_2_O_2_ exceeding 20 nM under nonstress conditions.^21^ Possibly, the concentration of H_2_O_2_ was not sufficient to saturate peroxidase activity and activate OxyR. Intriguingly, the gene product of the only upregulated OxyR target *grxA* (glutaredoxin-1) catalyzes reduction of activated OxyR via glutathione and therefore regulates OxyR in a negative feedback loop.^83^ Probably, other yet unknown transcription activators of *grxA* exist. Also, others have reported upregulation of the SoxRS but not the OxyR regulon under artificial oxidative stress conditions.^39,77,84^ Further investigations, for example, by means of proteomics or by measuring H_2_O_2_ levels, would be needed to shed more light on the observed results.

Gene ontology (GO) term enrichment analysis was performed to further explore the RNA-seq data. Among others (see Tables S2–S5), we identified the following enriched GO terms related to biological processes after 12 h of induction compared to the reference: “Cellular response to toxic substance” (GO: 0097237) in all Fab-producing strains, “Cellular response to oxidative stress” (GO: 0034599) in B⟨oBIBH1⟩ and B⟨oFTN2⟩, and “Response to oxidative stress” in BL21(DE3), B⟨oBIBH1⟩, and B⟨oFTN2⟩. Transcript levels of most genes within the three groups were downregulated, including the already described MnSOD (*sodA*). SodA is one of the three SODs in *E. coli* that contain different co-factors and are not functionally equivalent. Periplasmic CuZnSOD (*sodC*) which is expressed in an RpoS-dependent fashion in the stationary phase^85^ was downregulated as well in all strains including the BL21(DE3) wildtype (Figure S7). Within the enriched groups, *sodB* (coding for cytoplasmic FeSOD) was one of the few genes that showed expression upregulation. Transcript levels were increased in a Fab expression-dependent manner. Nevertheless, basal MnSOD and slightly increased FeSOD levels apparently were not sufficient to suppress SoxR activation during Fab expression.

### Downregulation of *nuo* and Upregulation of *ndh* in Fab-Producing Strains

**Figure 5:**
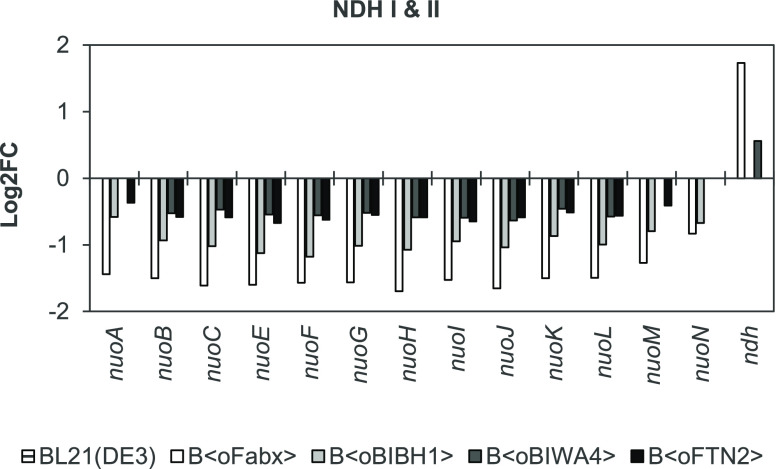
Log2FC of genes coding for NDH I (*nuo* operon) and NDH II (*ndh*) after 12 h of induction relative to the sample drawn prior to induction as determined by RNA-seq (α ≤ 0.05, *n* = 3).

GO term enrichment analysis revealed an impact of Fab expression on the respiratory chain, especially on expression of NDH I. We found that transcription of the *nuo* operon coding for subunit proteins of NDH I was downregulated after 12 h (Figure 5) and 16 h of induction (Figure S8). Log2FC varied from −0.3 (*nuoA* in B⟨oFTN2⟩) to −1.7 (*nuoH* in B⟨oFabx⟩) depending on the gene and strain. Fabx expression caused the most pronounced downregulation. Additionally, *ndh* (NDH II) transcript levels were increased in B⟨oFabx⟩ (log2FC of 1.7 at 12 h and 1.5 at 16 h) and B⟨oBIWA4⟩ (log2FC of 0.6 at 12 h). The wildtype reference also showed slight upregulation at 16 h. We observed no DGE of NDH genes at 2 h of induction. Since no data points were analyzed between 2 and 12 h, possible dynamics of *nuo* downregulation and *ndh* upregulation between these two time points are unknown.

During glucose-limited growth, the electron flux is directed through both NADH dehydrogenases in the presence of O_2_.^58^ Although both dehydrogenases oxidize NADH to NAD^+^, only NDH I contributes to the generation of the PMF (2 H^+^/e^–^). Therefore, the degree of coupling between electron transfer and H^+^ translocation (and hence ATP generation) depends on the distribution of e^–^ flux through NDH I and II (and between oxidases cytochrome bo and bd).^58,86^ Expression of *nuo* is influenced by growth conditions and ATP requirements.^87,88^ Growth impairment might have influenced downregulation of the operon in the strains producing the recombinant proteins. Since Fab productivity ceased toward the end of the process, ATP demand by recombinant protein production probably did as well. Downregulation of *nuo* and upregulation of *ndh* might indicate diverted electron flux from NDH I to NDH II. A change in usage of the two NADH dehydrogenases or rather increased activity of NDH II could have an implication for the formation of O_2_^•–^, since one of the sources of ROS is the autoxidizable flavin cofactor of NDH II.

## Conclusions

Fab’s are proteins that are challenging to produce in microbes, owing to various reasons. During periplasmic expression, factors such as translocation, folding (especially the necessity for disulfide bonds), and balancing expression between the two chains increase complexity and impact expression.^89,90^ Here, we identified additional, previously uncharacterized implications of periplasmic Fab expression in *E. coli*: (1) accumulation of superoxide and transcription activation of the oxidative stress-responsive gene *soxS* and (2) an insufficient ubiquinone availability to meet oxidative folding demand. Ubiquinone is used for electron transport in oxidative folding and in the respiratory chain, which is a major site of O_2_^•–^ formation; hence, the two observations are possibly connected and depend on process conditions. Oxidative stress and interference with the respiratory chain may have been involved in eliciting increased cell lysis and metabolic changes during Fab production in fed-batch processes, which impacted processability of the fermentation broth. A more detailed analysis, for example, of secreted metabolites would be desirable to further characterize shifts in energy metabolism. Within this study, we mainly focused on undesired O_2_^•–^ formation and ubiquinone deficiency. Under production conditions, increased O_2_^•–^ formation is an additional metabolic load and stress for the host cell. Oxidative stress was indicated by elevated transcript levels of *soxS* in all Fab production fed-batch processes which indicated activation of the SoxR transcription factor (presumably by O_2_^•–^). The source of O_2_^•–^ in our experiments remains unidentified. There are multiple possible candidates (including NDH II); therefore, it might be difficult to pinpoint a single source as the one responsible for the observed increased O_2_^•–^ levels. An RNA-seq analysis revealed that due to the glucose-limited growth conditions in our setup, different regulatory pathways were apparently counteracting (e.g., preventing increased expression of SoxS target *sodA* coding for ROS-scavenging MnSOD). Thereby, process conditions presumably impeded the cells’ capability to mitigate the harmful effects via the inducible oxidative stress response. This observation emphasizes the importance of applying relevant production conditions for comprehensive characterization of stress induced by recombinant protein expression (in our case, expression of Fab’s in a fed-batch process). Otherwise, conclusions drawn from small-scale batch experiments might not be valid under actual production conditions. The fact that supplementation of CoQ_1_ substantially improved Fab expression pointed toward ubiquinone shortage when the quinone pool is partitioned between dehydrogenases of the respiratory chain and oxidative folding. By artificially enhancing the available ubiquinone pool, for example, by supplementing UQ_8_ analogues, the cells’ disulfide bond formation capacity could be increased. The observation that the four studied Fab’s showed the same behavior albeit to a different extent can probably be explained by the heterodimeric nature of the model proteins. The HCs and LCs differ in sequence, which impacts expression and folding and dimerization of the chains and in further consequence accumulation of total recombinant protein. O_2_ consumption, O_2_^•–^ formation, and the achievable improvement of Fab yield by CoQ_1_ addition are not solely influenced by the correctly folded, soluble (quantifiable) Fab’s. Instead, the mixture of Fab, free chains, possible dimers, or other derivatives can vary in composition^46^ and has a level of impact that is hard to assess. Additionally, the different tendencies of the different Fab’s and Fab-derived molecules to aggregate (possibly even before disulfide bonds are formed) presumably play a part as well. Therefore, some questions remain to be studied in more detail, for example, by using additional, monomeric model proteins with varying numbers of disulfide bonds. Another intriguing possibility is the comparison between periplasmic and cytoplasmic expression of disulfide-containing proteins regarding the interactions between recombinant protein expression and the redox balance (e.g., ROS formation). Currently, several systems for the cytoplasmic production of disulfide bond-containing proteins in *E. coli* are available (such as SHuffle, Origami, and CysDisCo). These systems rely either on disruption of reducing pathways or co-expression of sulfhydryl oxidases and disulfide bond isomerases to facilitate oxidative folding within the usually reducing environment of the cytoplasm.^91^ In the future, our data could aid in the development of additional strategies to obtain *E. coli* strains capable of counteracting some of the negative effects of Fab expression, thereby providing better yields and processability through improved cell fitness.

## Materials and Methods

### Bacterial Strains

All used strains originated from *E. coli* BL21(DE3) (New England Biolabs (NEB), USA) and are listed in Table 1. Enzymes and kits for generation of the strains were purchased from NEB (USA). All constructs were confirmed by Sanger sequencing (Microsynth AG, Switzerland).

The design of the four different model Fab’s (Fabx, BIBH1, BIWA4, and FTN2), the construction of the integration cassettes, and the genome integration procedure of the constructs are described in detail in our previous work.^41^ Briefly, the Fab LCs and HCs were both fused to ompA^SS^ for post-translational translocation to the periplasm. LC and HC were expressed as bicistronic constructs, and each chain was equipped with its own ribosome-binding site. We used production systems with a single copy of the gene of interest integrated into the bacterial chromosome at the attTn7 site. Construction of the reference strain BL21(DE3) expressing GFPmut3.1 from a single copy integrated into the genome is described elsewhere.^92^ Since sfGFP exhibits fluorescence regardless of cellular localization, it was used as periplasmic reference protein. The co-translational dsbA^SS^ was used to mitigate cytosolic fluorescence. The dsbA^SS^-sfGFP construct was amplified from an in-house pET30a plasmid according to the same procedure as the Fab’s^41^ and integrated into the genome of BL21(DE3).^93^

### Media and Cultivation Conditions

#### Cell Banks

Master cell banks (MCBs) were prepared from cells grown in M9ZB medium. Baffled shake flasks were inoculated with a single colony and incubated at 37 °C and 180 rpm. Exponentially growing cultures were mixed 1:2 with 80% glycerol (Merck, Germany) when OD_600_ had reached 3.5 and aliquots were frozen at −80 °C. Working cell banks (WCBs) were inoculated from MCBs and grown in baffled shake flasks in semisynthetic medium (SSM). WCBs were cultured at 37 °C and 180 rpm. Like for the MCBs, cell aliquots were frozen in 40% glycerol at −80 °C after cultures had reached an OD_600_ of 3.5.

#### Shake Flask Cultivation

Shake flasks were grown in 25 mL of SSM in 250 mL baffled shake flasks. The cultures were inoculated from overnight cultures at on OD_600_ of 0.1 and grown at 37 °C and 180 rpm on an orbital shaker. Fab production was induced at an OD_600_ of 1.0 with 0.5 mM IPTG and the cultures were transferred to 30 °C. Cells were harvested after 4 h of production, and pellets equivalent to 1 mg CDM were frozen at −20 °C.

#### Fed-Batch-like Cultivation in a Microbioreactor System

Microscale cultivations were performed in the BioLector system (m2p-labs GmbH, Germany) as described by ref (41) with some modifications. Feed-in-time (FIT) medium containing 1 g L^–1^ glucose and 33 g L^–1^ EnPump200 dextran (Enpresso GmbH, Germany) was used in all experiments. Enzyme-mediated release of glucose (Carl Roth, Germany) was achieved by the addition of 0.6% (v v^–1^) EnzMix (Enpresso GmbH, Germany). FIT medium was supplemented with (1) 148.3 mM MOPS, (2) 1.7 μM CoCl_2_·6H_2_O, (3) 56.1 mM (NH_4_)_2_SO_4_, (4) 12.8 mM K_2_HPO_4_, (5) 7.6 mM Na_3_Citrate·2H_2_O, (6) 3.1 M MgSO_4_·7H_2_O, (7) 1.4 μM ZnSO_4_·7H_2_O, (8) 114.4 μM FeCl_3_·6H_2_O, (9) 10.4 mM Na_2_SO_4_, (10) 22.0 μM Thiamine·HCl, (11) 1.5 μM CuSO_4_·5H_2_O, (12) 1.3 μM MnSO_4_·H_2_O, (13) 66.5 μM Titriplex III, (14) 13.9 mM NH_4_Cl, and (15) 10.1 μM CaCl_2_·2H_2_O. (1) and (2) were purchased from Sigma-Aldrich, USA, (3–8) from Carl Roth, Germany, (9–13) from Merck, Germany, and (14) and (15) from Applichem, Germany. 48-well flower plates (m2p-labs GmbH, Germany) with a working volume of 800 μL were used. The cultures were inoculated from WCBs with an initial OD_600_ of 0.3. Temperature was maintained at 30 °C, the shaking frequency at 1400 rpm, and the humidity at >85%. Biomass accumulation was analyzed as described by ref (41). Additionally, biomass of endpoint samples was determined gravimetrically. Fab production was induced with 0.5 mM IPTG (GERBU Biotechnik, Germany) after 9 h of cultivation. Endpoint samples were drawn 12 h after induction.

#### CoQ_1_ Supplementation

CoQ_1_ was supplemented to shake flask cultivations or selected wells of microtiter cultivations at induction. CoQ_1_ was obtained from Sigma-Aldrich, USA, and a 200 mM stock solution was prepared in acetone. The stock was further diluted to 100× the final concentrations in 50% ethanol and the respective amount added to the cultivations.

#### Fed-Batch Cultivation

Fed-batch cultivations were conducted in a DASGIP Parallel Bioreactor System (Eppendorf AG, Germany) as described by ref 94^94^ with 0.6 L batch volume and 1.25 L final working volume. Reactors were equipped with standard control units and a GA4X-module (Eppendorf AG, Germany) for off-gas analysis. Temperature was maintained at 37 ± 0.5 °C during the batch phase and was decreased to 30 ± 0.5 °C at the beginning of the feeding phase. The pH was kept constant at 7.00 ± 0.05 by the addition of 12.5% ammonia solution (w w^–1^). DO was set to 30% and maintained by adjusting the stirrer speed, aeration rate, and in-gas composition. The batch was inoculated from precultures according to ref (94). Cells were grown to 6 g in the batch phase after which the exponential carbon-limited feed was started. The growth rate was controlled at μ = 0.1 h^–1^. Recombinant protein production was induced after 3 h of feed with 2 μmol IPTG g^–1^ CDM. Production was continued for 16 h (approx. 2 generations) resulting in a theoretical biomass of 40 g. Components for batch and fed-batch media were obtained from Carl Roth, Germany. Media were prepared gravimetrically according to final biomass. Glucose was added to batch and fed-batch media according to the theoretical final biomass based on a yield coefficient of *Y*_X/S_ = 0.3 g/g. Compositions of batch and fed-batch media are described elsewhere.^94^ To prevent the formation of foam, PPG2000 antifoam (BASF, Germany) was added on demand. Cultivations used for RNA-seq were conducted in triplicate.

### Offline Analytics

#### Biomass Quantification

CDM from fed-batch cultivations was determined gravimetrically as described by ref (94).

#### Sampling and Cell Disruption

To analyze recombinant protein production, cell aliquots corresponding to 1 mg of CDM were sampled. Endpoint samples were drawn from shake flask and microscale cultivations. Samples from fed-batch fermentations were drawn every 2 h. The aliquots were pelleted (10 min, 13,000*g*) and frozen at −20 °C. Total protein was extracted enzymatically as described by ref (41).

Samples for RNA-seq and qPCR were drawn from fed-batch cultivation preinduction and after 2, 12, and 16 h of induction. Sampled cells were immediately transferred into 0.5× the volume of a 5% phenol in ethanol solution on ice. 3 mg CDM aliquots were spun down at 4 °C and 13,000*g* for 2 min and stored at −80 °C.

#### Analysis of Recombinant Protein by WB

Expression of Fab and LCs in the soluble and IB fraction of cell lysates and in the culture supernatant were analyzed by LC-specific WB analysis using anti-human κ-LC (bound and free) goat antibody, conjugated to alkaline phosphatase (A3813; Sigma-Aldrich, USA) as described by ref (41).

#### Quantification of Fab by ELISA

Fab in the soluble fraction of cell extracts and the culture supernatant was quantified using a sandwich ELISA as described by ref (41).

#### Quantification of GFP by Fluorometry Analysis

GFP was quantified using a Tecan analyzer infinite 200Pro (485/520 nm) and a calibration curve constructed with in-house-purified GFP as previously described.^92^

#### Flow Cytometric Superoxide Measurement

Intracellular superoxide levels were measured flow cytometrically using CellROX Green reagent (Invitrogen, USA). Cells were sampled from the respective cultivations and diluted to a final OD_600_ of 0.035 in 1× PBS. CellROX Green reagent was thawed, diluted in 1× PBS (flow cytometry grade), and added to the cells at a final concentration of 4 μM. The optimal ratio of cell to dye concentration (114× CellROX Green) had been determined in preliminary experiments and was defined as the concentration at which a maximal signal was obtained, without using excessive amounts of dye. The cells were incubated at 37 °C for 30 min with gentle mixing for staining. Once thawed, the CellROX Green reagent aliquots were protected from light and used within 2 h. Cells treated with 350 μM MD served as a positive control. MD treatment was included during CellROX Green staining. Samples were measured on a CytoFLEX S flow cytometer (Beckman Coulter, USA). CellROX Green reagent was excited by a 488 nm laser and emission detected with a 525/40 nm band pass filter. The flow rate was set to 60 μL min^–1^, and 15,000 events were collected for each sample. Samples were analyzed in biological triplicate. The obtained data were analyzed using the CytExpert Software (Beckman Coulter, USA). *E. coli*-sized particles were gated in FSC/SSC plots to remove small particles and cell debris (Figure S9).

### Gene Expression Analysis

#### RNA Extraction

Cell pellets were thawed, and cells were disrupted for 10 min with 10 mg mL^–1^ lysozyme in TE-buffer (1 mM EDTA, 10 mM Tris–HCl) while vortexing. Then, TRIzol reagent (Invitrogen, USA) was added to a final cell count of approx. 1.5 × 10^8^ and the samples were incubated for another 5 min on the vortex. RNA was extracted using a Direct-zol RNA Miniprep Kit (Zymo Research, USA) according to the manufacturer’s instructions. An equal volume of ethanol was added to the samples in TRIzol. The samples were transferred to a Zymo-Spin Mini column, spun down at 13,400*g* for 30 s, and washed. To remove DNA, the samples were treated with DNase I for 30 min according to the kit manual. After three washing steps, the RNA was eluted in 25 μL of nuclease-free water. Quantification of total RNA and assessment of protein and phenol contamination was performed using a NanoDrop ND-1000 (Thermo Fisher, USA). RNA integrity and the absence of genomic DNA were analyzed with an Agilent 2100 Bioanalyzer using the RNA 6000 Nano Kit (Agilent Technologies, USA). Only samples with an RNA integrity number (RIN) > 8 were used. RNA extracts were stored at −80 °C.

#### RNA-Seq Library Preparation and Sequencing

Ribosomal RNA removal was performed with the Ribo-Zero rRNA Removal Kit Bacteria (Illumina, USA). Sequencing libraries of the rRNA depleted samples were prepared using the NEBNext Ultra II Directional RNA Library Prep Kit for Illumina (New England Biolabs, USA). Libraries were sequenced in single-read mode on a HiSeq 2500 system (Illumina, USA). rRNA depletion, library preparation, and sequencing were performed by the Next Generation Sequencing Facility at Vienna BioCenter Core Facilities (VBCF), member of the Vienna BioCenter (VBC), Austria.

#### Sequencing Read Preprocessing and Mapping

The quality and the adapter content of the raw RNA-seq reads were analyzed with FastQC.^95^ Then, the raw reads were trimmed with Trimmomatic v0.38^96^ to remove adapters and low-quality reads. Reads with a Phred quality score ≥ 25 and a length ≥ 35 bp after trimming were kept for further analysis. The trimming was conducted providing the NEBNext adapter sequence as a template to assess and remove any adapter content. The quality and the adapter content of the trimmed reads were then re-assessed with FastQC and a summary was obtained with MultiQC.^97^ The quality-trimmed RNA-seq reads from each sample were mapped onto the corresponding reference genome. The reference genome of *E. coli* BL21(DE3) used for mapping of the RNA-seq reads had been previously determined in-house by whole genome sequencing.^92^ The mapping was conducted with HISAT2^98^ using the following adapted parameters: --score-min L,0.0,-0.2 --no-spliced-alignment --no-softclip. The remaining parameters of the program were left as default. The BAM files resulting from the mapping were filtered with samtools^99^ removing unmapped reads and secondary alignments (-F0x4-F0x0100). The filtered BAM files were sorted and indexed with samtools.

#### DGE Analysis

The filtered, sorted, and indexed BAM files were used together with the GFF annotation of the reference genome to count read occurrences at each gene with HTSeq-count.^100^ The following parameters were used: --format bam --order pos --stranded reverse --minaqual 20 --type exon --mode union --secondary-alignments ignore --supplementary-alignments ignore --idattr Name. Read counts per gene produced by HTSeq for each sample were merged into a single table of counts with a custom python script. The table of counts was used as input to calculate differentially expressed genes (DEGs) with DESeq2.^101^ Genes with an average of less than 10 counts across all replicates were excluded from the analysis. A normalized distribution of counts was obtained with samples within the function DESeq(sfType = “poscounts”). DEGs were computed within the same strains at different time points, comparing 2, 12, and 16 h postinduction samples against their corresponding noninduced (0 h) samples. DEGs were calculated with the function res(alpha = 0.05, altHypothesis = “greaterAbs”, lfcThreshold = 0.1). Computed log2FoldChanges were reduced using the function lfcShrink(type = “ashr”). Genes showing a log2FC ≥ 0.1 (in absolute value) and a *p*-value ≤ 0.05 were considered differentially expressed and statistically significant.

#### GO Term Analysis

The potential enrichment of any GO term^102^ in the DEGs was assessed in a custom R script with ClusterProfiler.^103^ In each sample, the enrichment of the GO terms associated to each DEG (i.e., “Selection”) was compared to their abundance in the complete *E. coli* gene set (i.e., “Universe”, source: ecocyc). The library “org.EcK12.eg.db” was used to convert gene aliases to Entrez Gene IDs using the function “org.EcK12.egALIAS2EG”. Enriched GO Terms were extracted using the function enrichGO(), targeting biological process (“BP”), molecular function (“MF”), and cellular component (“CC”) terms in three separate runs. The complete function arguments were declared as follows: enrichGO(Selection, org.EcK12.eg.db, keyType = “ENTREZID”, ont = “BP”, pvalueCutoff = 0.05, pAdjustMethod = “BH”, Universe, qvalueCutoff = 0.05, minGSSize = 15, maxGSSize = 500, readable = TRUE, pool = FALSE). The “ont” argument was changed to MF and CC according to the type of GO term assessed. Resulting enriched GO terms were simplified merging similar GO terms using the simplify() function, with a similarity cutoff of 0.8. Simplified enriched GO terms were then filtered using the function gofilter(level = ...). For each of the three runs (BP, MF, and CC), four independent filtering runs were generated, each at a different GO term level (3, 4, 5, and 6), representing increasing depths of GO term characterization.

#### Reverse Transcription

Approx. 1.5 μg of total RNA was reverse-transcribed to cDNA using Superscript III Reverse Transcriptase (Invitrogen, USA) in 20 μL reaction volume according to the manufacturer’s instructions. 230 ng of random hexamer primers was used; hence, the reaction mixtures were incubated for 10 min at room temperature. Reverse transcription was performed at 50 °C for 50 min. 40 U of RNasin Ribonuclease Inhibitor (Promega, USA) were added to the reaction mixtures. No reverse transcriptase controls were prepared for all samples by replacing Superscript III Reverse Transcriptase with nuclease-free water. Reverse-transcribed samples were treated with 5 U RNase H (NEB, USA) for 20 min at 37 °C to remove the RNA template. A Qubit 4 fluorometer was used to quantify cDNA with a Qubit dsDNA HS Assay Kit (Invitrogen, USA) according to the manufacturer’s instructions. Samples were stored at −20 °C.

#### qPCR

Efficiencies and melting curves of selected primer pairs (Table S6, purchased from Sigma-Aldrich, USA) binding to transcripts of the target *soxS* and the reference gene *cysG* were evaluated using fivefold dilution series of the pooled samples as a template (Figure S10). Stability of *cysG* transcript levels and similar expression between all tested strains had been previously determined by RNA-seq (Figure S11). 2× iQ SYBR Green Supermix (Bio-Rad, USA) was used according to manufacturer’s instructions. Primers were diluted to a final concentration of 300 nM. Approx. 1 ng of cDNA samples was used in a reaction volume of 20 μL. No template controls were included. The PCRs were carried out in white 48-well PCR plates (Bio-Rad, USA) in a MiniOpticon Real-Time PCR System (Bio-Rad, USA). Thermocycling included an initial denaturation and enzyme activation step (95 °C, 3 min), 39 cycles of denaturing (95 °C, 10 s), annealing (62 °C, 30 s), and extension (72 °C, 30 s), and determination of the melt curve (55–95 °C in 0.5 °C increments with 20 s holding time). All cDNA samples were measured in triplicate. CFX Manager Software (Bio-Rad, USA) was used for data analysis. Quantification cycles (Cq) were determined in the single threshold mode (automatic). Expression of the target gene was normalized to the reference gene according to the ΔΔCq method. The noninduced sample was used as a reference.

## Supporting Information Available

% of total soluble Fab found extracellularly in the culture supernatant in fed-batch fermentations; Fab fragment expression patterns (LC-specific WB) obtained by fed-batch-like microtiter cultivations that were used for flow cytometric measurement of O_2_^•–^; histograms and FSC/SSC plots of flow cytometric measurements of B⟨oFabx⟩, B⟨oBIBH1⟩ and B⟨oBIWA4⟩ cells obtained from fed-batch-like microtiter cultivations; LC-specific WB of B⟨oFTN2⟩ shake flask cultivations without and with 5 μM CoQ_1_ supplementation; log2FC of differentially expressed members of the SoxRS and OxyR regulons 16 h postinduction as determined by RNA-seq; O_2_ [%] in the in-gas stream of fed-batch cultivations and log2FC of *soxS* transcript levels; log2FC of *sodB* and *sodC* in fed-batch cultivations; log2FC of the *nuo* operon, *ndh* and the *ubi* genes 16 h postinduction; gating of the *E. coli* population in flow cytometry data; efficiencies and melting peaks of primers used for *soxS* qPCR; and *cysG* transcript levels in fed-batch cultivations obtained by RNA-seq (PDF)
Transcriptomic data are available under the SRA BioProject PRJNA750301 (PDF)

## Abbreviations

CDM: cell dry mass
CoQ_1_: coenzyme Q1
DEGs: differentially expressed genes
DGE: differential gene expression
DMQ: demethylmenaquinone
DO: dissolved oxygen
*E. coli*: *Escherichia coli*
ELISA: enzyme-linked immunosorbent assay
ER: endoplasmic reticulum
Fab: fragment antigen binding
FIT: feed-in-time
FSC: forward scatter
(GeoMean) FI: (geometric mean of) fluorescence intensity
GFP: green fluorescent protein
GO: gene ontology
HC: heavy chain
IB: inclusion body
IM: inner membrane
IPTG: β-d-1-thiogalactopyranoside
LC: light chain
log2FC: log2 fold change
MD: menadione
MQ: menaquinone
NDH I/II: NADH dehydrogenase I/II
OD: optical density
ompA^SS^: ompA signal sequence
PMF: proton motive force
qCO_2_: CO_2_ formation rate
qO_2_: O_2_ consumption rate
RNA-seq: RNA sequencing
ROS: reactive oxygen species
SSC: side scatter
SSM: semisynthetic medium
UQ_8_: ubiquinone
WB: western blot
WCB: working cell bank
