# Supplementary material for: Interaction of Periplasmic Fab Production and Intracellular Redox Balance in Escherichia coli Affects Product Yield
Source: ACS Synth Biol. 2022 Jan 18;11(2):820–34. doi: 10.1021/acssynbio.1c00502 (PMC8859853; doi:10.1021/acssynbio.1c00502)

Interaction of periplasmic Fab production and intracellular redox balance in *Escherichia coli* affects product yield

Sophie Vazulka[1], Matteo Schiavinato[2], Martin Wagenknecht[3], Monika Cserjan-Puschmann[1], Gerald Striedner[1]

1 Christian Doppler Laboratory for production of next-level biopharmaceuticals in E. coli, University of Natural Resources and Life Sciences, Vienna, Department of Biotechnology, Institute of Bioprocess Science and Engineering, Muthgasse 18, 1190 Vienna, Austria
2 University of Natural Resources and Life Sciences, Vienna, Department of Biotechnology, Institute of Computational Biology, Muthgasse 18, 1190 Vienna, Austria
3 Boehringer Ingelheim RCV GmbH & Co KG, Dr.-Boehringer-Gasse 5-11, 1120 Vienna, Austria

Figure S1. % of the total, soluble Fab [mg g^{-1}] found extracellularly in the culture supernatant over the course of fed-batch fermentations. Induction is indicated by the dashed grey line.

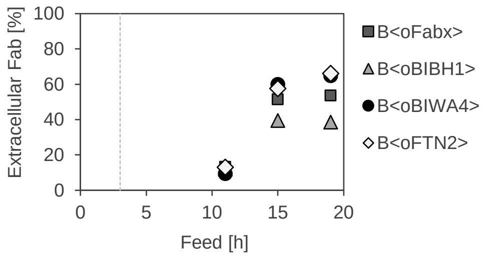

Figure S2. Fab fragment expression patterns obtained by fed-batch-like microtiter cultivations that were used for flow cytometric measurement of $O_2^{\bullet-}$. For comparability of the LC-specific WBs the loaded samples were adjusted to the same biomass. Soluble (1) and IB fractions (2) of cell lysates are shown.

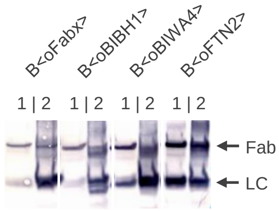

1 ... Soluble fraction
2 ... Inclusion body

Figure S3A. Histograms of BL21(DE3) strains producing Fabx, BIBH1 and BIWA4 obtained from fed-batch-like microtiter cultivations after 12 h of cultivation without (0 mM IPTG) and with induction (0.5 mM IPTG) and without (–CG) and with CellROX Green staining (+CG). The positive control (BL21(DE3) treated with 350 µM MD) is shown in all graphs.

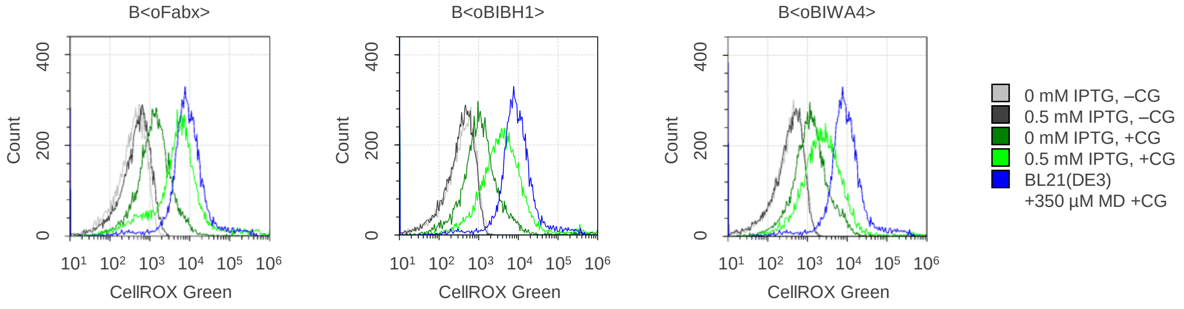

Figure S3B. FSC/SSC plot of B<oFabx> with and without induction.

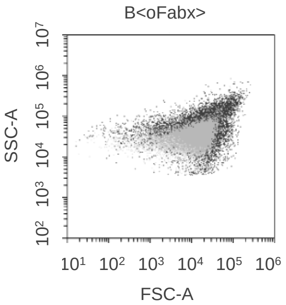

Figure S3C. FSC/SSC plot of B<oBIBH1>, B<oBIWA4> and B<oFTN2> with and without induction. The noninduced sample is shown with and without CellROX Green staining.

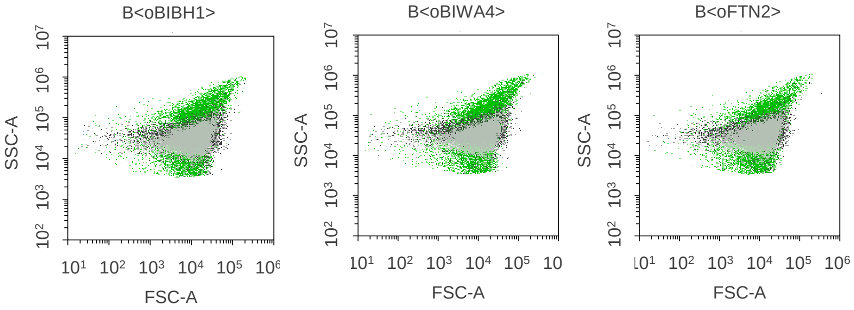

Figure S4. LC-specific WB of B<oFTN2> shake flask cultivations without and with 5 µM CoQ1 supplementation. Soluble (1) and IB fractions (2) and the culture supernatant (3) are shown. Samples were adjusted to the same biomass in each lane for comparability.

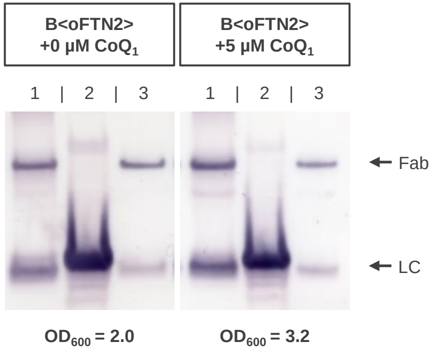

1 ... Soluble fraction
2 ... Inclusion bodies
3 ... Extracellular fraction

Figure S5. Log2FC of differentially expressed members of the SoxRS and OxyR regulons 16 h post-induction relative to the noninduced sample as determined by RNA-seq (n = 3, α ≤ 0.05) for the Fab production strains and the wildtype reference BL21(DE3).

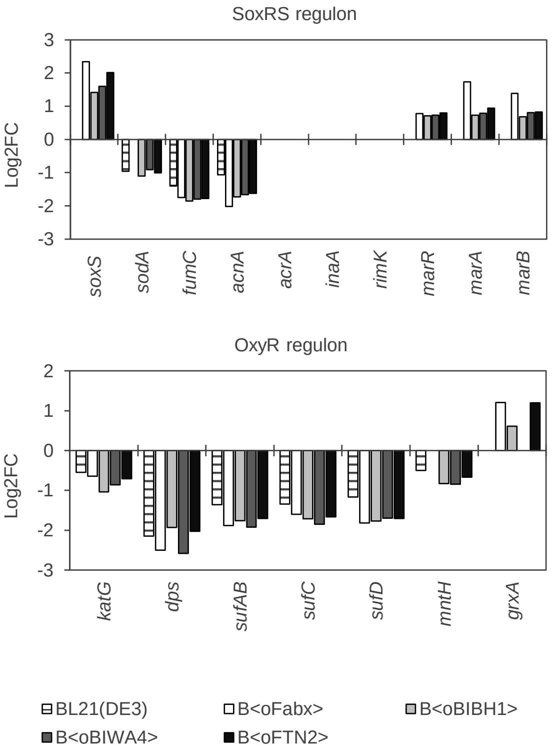

Figure S6A. O_2 in the in-gas stream [%] of fed-batch cultivations and log2FC of *soxS* transcript levels as determined by RNA-seq (n = 3, α ≤ 0.05) over the course of fed-batch cultivations. A single representative cultivation is shown for the O_2-enrichment of process air. Induction is indicated by the dashed grey line.

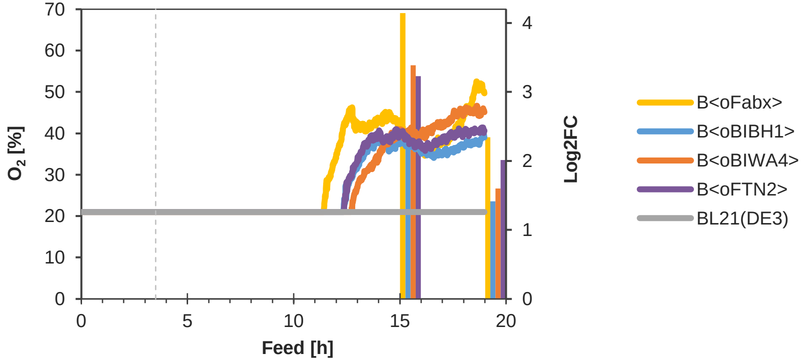

Figure S6B. Δ Δ Cq normalized gene expression of *soxS* in B<oFTN2> during the course of a fed-batch cultivation as measured by qPCR in technical triplicate (n = 3).

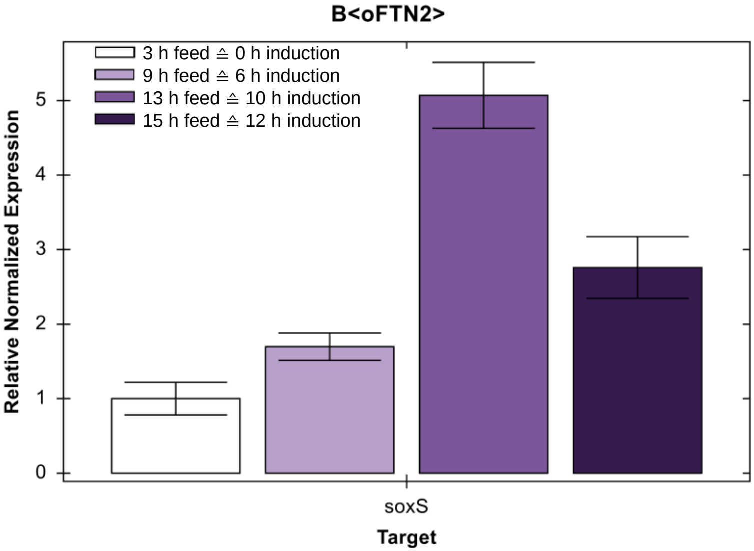

Figure S7. Log2FC of *sodB* and *sodC* in fed-batch cultivations relative to the noninduced sample as determined by RNA-seq (n = 3, $\alpha \leq 0.05$). Samples of the Fab production strains and the wildtype reference BL21(DE3) drawn after 2, 12 and 16 h of induction are shown.

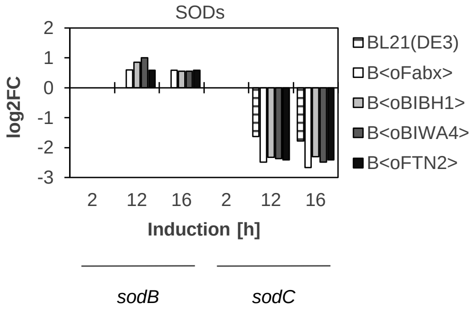

Figure S8. Log2FC of the *nuo* operon, *ndh* and the *ubi* genes 16 h post-induction as determined by RNA-seq (n = 3, α ≤ 0.05) for the Fab production strains and the BL21(DE3) wildtype reference strain.

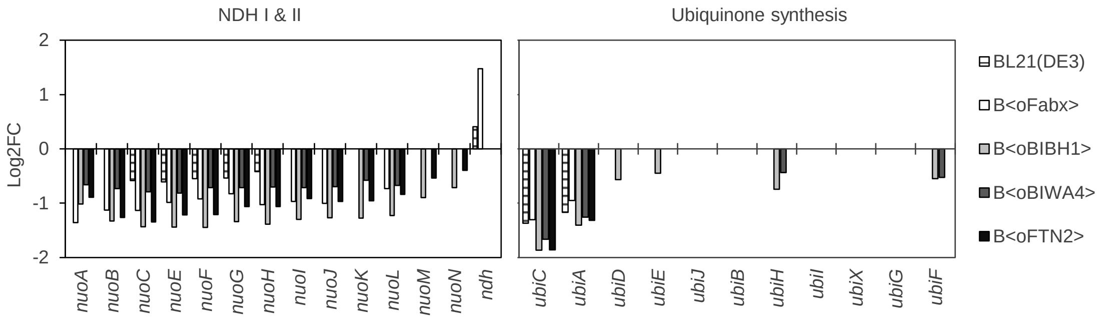

Figure S9. Gating of the *E. coli* population in flow cytometry data. A culture of the BL21(DE3) wildtype reference with induction is shown without and with CellROX Green staining.

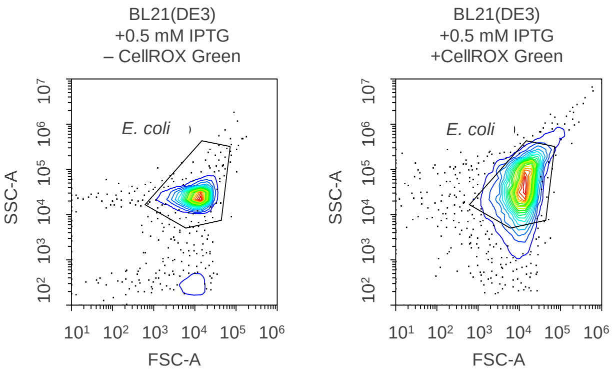

Figure S10. Standard curves (including primer efficiencies) and melting peaks obained by qPCR using primers targeting *soxS* and *cysG*. All cDNA samples to be analyzed were pooled and measured in triplicate.

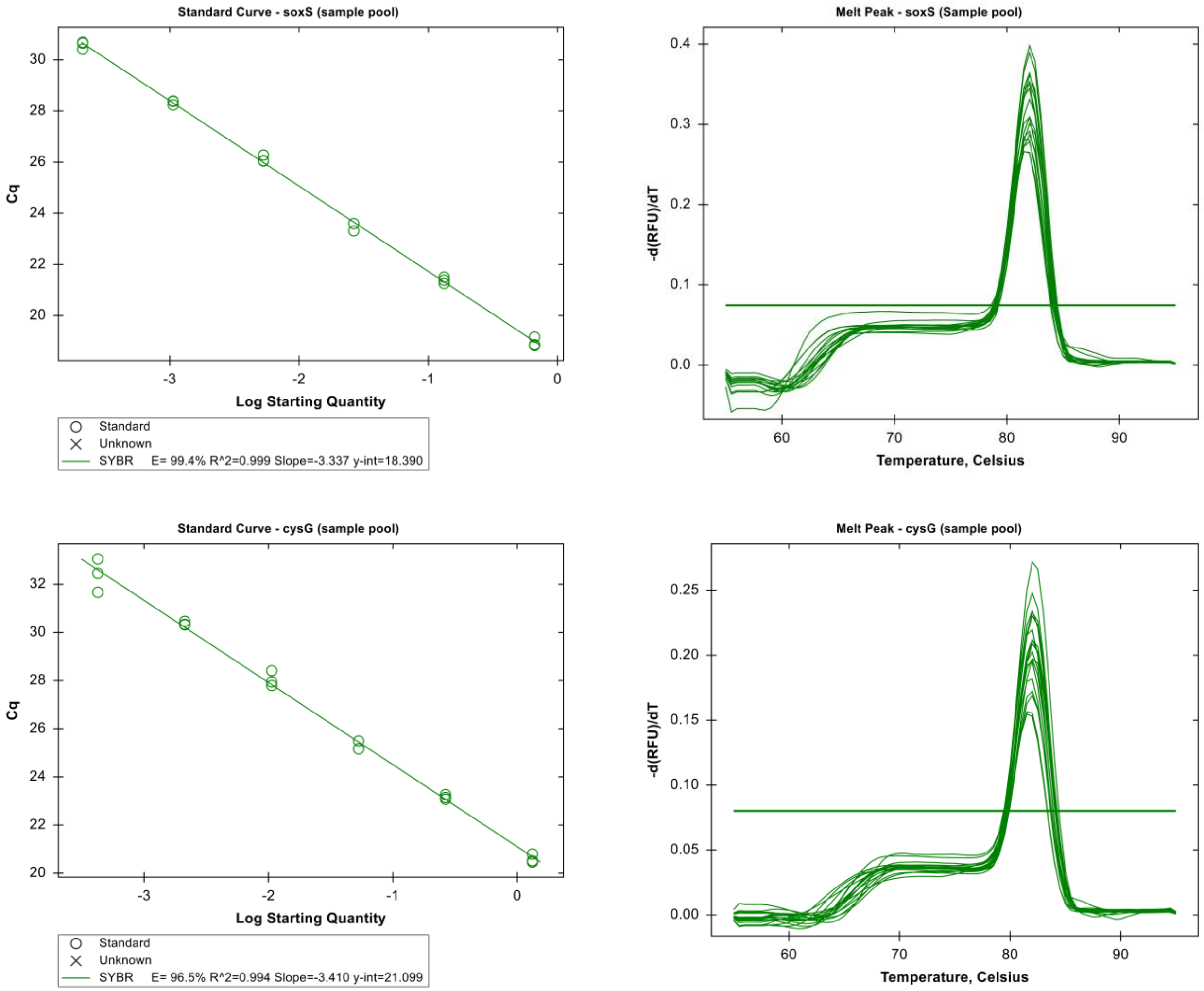

Figure S11. Transcript levels in transcripts per million (TPM) of *cysG* during fed-batch cultivations of Fab producers and the BL21(DE3) wildtype reference. DGE analysis (DESeq2) showed no significant (α = 0.05) changes in *cysG* expression in any of the tested strains at any timepoint relative to the noninduced sample (0 h). Samples were analyzed in biological triplicate (n = 3, Mean + SEM).

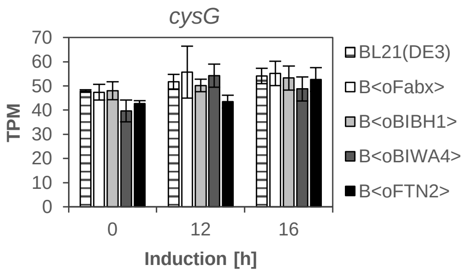

Supplement: Supplementary file 1 — sb1c00502_si_001.pdf [file sb1c00502_si_001.pdf]
