# Supplementary material for: Interaction of Periplasmic Fab Production and Intracellular Redox Balance in Escherichia coli Affects Product Yield
Source: ACS Synth Biol. 2022 Jan 18;11(2):820–34. doi: 10.1021/acssynbio.1c00502 (PMC8859853; doi:10.1021/acssynbio.1c00502)
Supplement: Supplementary file 2 — sb1c00502_si_002.pdf [file sb1c00502_si_002.pdf]

Interaction of periplasmic Fab production and intracellular redox balance in *Escherichia coli* affects product yield

Sophie Vazulka[1], Matteo Schiavinato[2], Martin Wagenknecht[3], Monika Cserjan-Puschmann[1]*, Gerald Striedner[1]

1 Christian Doppler Laboratory for production of next-level biopharmaceuticals in *E. coli*, University of Natural Resources and Life Sciences, Vienna, Department of Biotechnology, Institute of Bioprocess Science and Engineering, Muthgasse 18, 1190 Vienna, Austria

2 University of Natural Resources and Life Sciences, Vienna, Department of Biotechnology, Institute of Computational Biology, Muthgasse 18, 1190 Vienna, Austria

3 Boehringer Ingelheim RCV GmbH & Co KG, Dr.-Boehringer-Gasse 5-11, 1120 Vienna, Austria

Table S1: Selection of genes differentially expressed in fed-batch cultivations after 12 h of induction (n = 3, α ≤ 0.05).

Gene	Strain	Log2FC	P value
ubiC	BL21(DE3)	-1.06	$6.31*10^{-16}$
	B<oFabx>	-1.17	$1.91*10^{-7}$
	B<oBIBH1>	-1.31	$1.05*10^{-10}$
	B<oBIWA4>	-0.89	$3.25*10^{-6}$
	B<oFTN2>	-1.24	$4.17*10^{-12}$
ubiA	BL21(DE3)	-0.81	$2.24*10^{-12}$
	B<oFabx>	-0.89	$2.00*10^{-4}$
	B<oBIBH1>	-0.92	$2.07*10^{-6}$
	B<oBIWA4>	-0.74	$1.77*10^{-6}$
	B<oFTN2>	-0.93	$8.06*10^{-10}$
soxS	B<oFabx>	4.15	$4.98*10^{-52}$
	B<oBIBH1>	2.36	$1.07*10^{-16}$
	B<oBIWA4>	3.39	$1.96*10^{-67}$
	B<oFTN2>	3.23	$1.50*10^{-26}$
sodA	BL21(DE3)	-0.90	$3.19*10^{-8}$
	B<oFabx>	0.56	$7.51*10^{-3}$
	B<oBIBH1>	-0.78	$2.14*10^{-3}$
	B<oBIWA4>	-0.48	$3.18*10^{-3}$
	B<oFTN2>	-0.91	$2.14*10^{-4}$
acrA	B<oFabx>	0.58	$1.18*10^{-2}$
inaA	B<oFabx>	0.70	$2.66*10^{-3}$
rimK	B<oFabx>	0.82	$4.64*10^{-4}$
fumC	BL21(DE3)	-0.92	$3.30*10^{-14}$
	B<oFabx>	-1.34	$5.73*10^{-9}$
	B<oBIBH1>	-1.53	$6.77*10^{-11}$
	B<oBIWA4>	-1.25	$9.98*10^{-16}$
	B<oFTN2>	-1.43	$1.02*10^{-10}$
acnA	BL21(DE3)	-0.86	$1.56*10^{-16}$
	B<oFabx>	-1.91	$1.16*10^{-14}$
	B<oBIBH1>	-1.62	$1.94*10^{-18}$
	B<oBIWA4>	-1.42	$1.28*10^{-24}$
	B<oFTN2>	-1.55	$2.55*10^{-29}$
marR	B<oFabx>	2.75	$1.73*10^{-18}$
	B<oBIBH1>	1.40	$4.23*10^{-5}$
	B<oBIWA4>	1.78	$2.52*10^{-16}$
	B<oFTN2>	1.65	$2.21*10^{-10}$
marA	B<oFabx>	3.06	$2.34*10^{-26}$
	B<oBIBH1>	1.18	$7.80*10^{-6}$
	B<oBIWA4>	1.87	$3.57*10^{-30}$
	B<oFTN2>	1.19	$3.64*10^{-9}$

Interaction of periplasmic Fab production and intracellular redox balance in *Escherichia coli* affects product yield

Sophie Vazulka[1], Matteo Schiavinato[2], Martin Wagenknecht[3], Monika Cserjan-Puschmann[1]*, Gerald Striedner[1]

1 Christian Doppler Laboratory for production of next-level biopharmaceuticals in *E. coli*, University of Natural Resources and Life Sciences, Vienna, Department of Biotechnology, Institute of Bioprocess Science and Engineering, Muthgasse 18, 1190 Vienna, Austria

2 University of Natural Resources and Life Sciences, Vienna, Department of Biotechnology, Institute of Computational Biology, Muthgasse 18, 1190 Vienna, Austria

3 Boehringer Ingelheim RCV GmbH & Co KG, Dr.-Boehringer-Gasse 5-11, 1120 Vienna, Austria

marB	B<oFabx>	2.79	$1.32*10^{-16}$
	B<oBIWA4>	1.79	$9.60*10^{-18}$
	B<oFTN2>	1.01	$6.13*10^{-4}$
ybjC	B<oFabx>	1.04	$1.85*10^{-4}$
katG	BL21(DE3)	-0.34	$2.57*10^{-3}$
	B<oFabx>	-0.71	$1.24*10^{-3}$
	B<oBIBH1>	-0.88	$4.52*10^{-5}$
	B<oBIWA4>	-0.52	$1.29*10^{-3}$
	B<oFTN2>	-0.72	$2.00*10^{-7}$
dps	BL21(DE3)	-2.03	$7.99*10^{-60}$
	B<oFabx>	-2.87	$5.45*10^{-30}$
	B<oBIBH1>	-2.42	$7.98*10^{-20}$
	B<oBIWA4>	-2.95	$1.94*10^{-109}$
	B<oFTN2>	-2.88	$1.17*10^{-72}$
sufAB	BL21(DE3)	-1.32	$2.07*10^{-25}$
	B<oFabx>	-1.17	$2.14*10^{-6}$
	B<oBIBH1>	-1.67	$2.95*10^{-15}$
	B<oBIWA4>	-1.67	$7.25*10^{-27}$
	B<oFTN2>	-1.86	$1.25*10^{-26}$
sufC	BL21(DE3)	-1.04	$2.35*10^{-13}$
	B<oFabx>	-1.25	$4.34*10^{-7}$
	B<oBIBH1>	-1.66	$1.07*10^{-15}$
	B<oBIWA4>	-1.67	$6.05*10^{-28}$
	B<oFTN2>	-1.54	$3.80*10^{-22}$
sufD	BL21(DE3)	-1.02	$4.70*10^{-16}$
	B<oFabx>	-1.40	$2.76*10^{-10}$
	B<oBIBH1>	-1.58	$5.45*10^{-16}$
	B<oBIWA4>	-1.54	$1.55*10^{-23}$
	B<oFTN2>	-1.64	$1.86*10^{-20}$
mntH	BL21(DE3)	-0.63	$2.20*10^{-6}$
	B<oFabx>	-1.26	$1.96*10^{-8}$
	B<oBIBH1>	-1.14	$8.54*10^{-10}$
	B<oBIWA4>	-1.42	$1.50*10^{-20}$
	B<oFTN2>	-1.24	$2.76*10^{-14}$
grxA	B<oFabx>	1.47	$9.85*10^{-7}$
	B<oBIBH1>	0.82	$8.06*10^{-3}$
	B<oBIWA4>	0.86	$8.91*10^{-5}$
	B<oFTN2>	1.04	$5.80*10^{-4}$
sodB	B<oFabx>	0.60	$8.14*10^{-3}$
	B<oBIBH1>	0.85	$3.10*10^{-4}$
	B<oBIWA4>	1.00	$5.73*10^{-12}$
	B<oFTN2>	0.59	$8.71*10^{-5}$
sodC	BL21(DE3)	-1.63	$4.12*10^{-32}$

Sophie Vazulka[1], Matteo Schiavinato[2], Martin Wagenknecht[3], Monika Cserjan-Puschmann[1]*, Gerald Striedner[1]

1 Christian Doppler Laboratory for production of next-level biopharmaceuticals in *E. coli*, University of Natural Resources and Life Sciences, Vienna, Department of Biotechnology, Institute of Bioprocess Science and Engineering, Muthgasse 18, 1190 Vienna, Austria

2 University of Natural Resources and Life Sciences, Vienna, Department of Biotechnology, Institute of Computational Biology, Muthgasse 18, 1190 Vienna, Austria

3 Boehringer Ingelheim RCV GmbH & Co KG, Dr.-Boehringer-Gasse 5-11, 1120 Vienna, Austria

	B<oFabx>	-2.48	$3.96*10^{-27}$
	B<oBIBH1>	-2.33	$3.53*10^{-26}$
	B<oBIWA4>	-2.37	$3.94*10^{-57}$
	B<oFTN2>	-2.41	$1.42*10^{-55}$
nuoA	B<oFabx>	-1.44	$5.18*10^{-7}$
	B<oBIBH1>	-0.58	$2.00*10^{-3}$
	B<oFTN2>	-0.37	$8.19*10^{-3}$
nuoB	B<oFabx>	-1.50	$6.65*10^{-8}$
	B<oBIBH1>	-0.93	$6.57*10^{-7}$
	B<oBIWA4>	-0.52	$1.67*10^{-3}$
	B<oFTN2>	-0.58	$1.06*10^{-4}$
nuoC	B<oFabx>	-1.61	$6.75*10^{-7}$
	B<oBIBH1>	-1.02	$2.08*10^{-9}$
	B<oBIWA4>	-0.47	$8.00*10^{-3}$
	B<oFTN2>	-0.59	$1.58*10^{-3}$
nuoE	B<oFabx>	-1.60	$4.64*10^{-7}$
	B<oBIBH1>	-1.12	$6.13*10^{-10}$
	B<oBIWA4>	-0.54	$1.70*10^{-3}$
	B<oFTN2>	-0.67	$8.21*10^{-6}$
nuoF	B<oFabx>	-1.57	$6.52*10^{-7}$
	B<oBIBH1>	-1.18	$1.03*10^{-11}$
	B<oBIWA4>	-0.55	$2.16*10^{-3}$
	B<oFTN2>	-0.62	$7.68*10^{-5}$
nuoG	B<oFabx>	-1.57	$3.35*10^{-7}$
	B<oBIBH1>	-1.02	$3.06*10^{-8}$
	B<oBIWA4>	-0.52	$3.90*10^{-3}$
	B<oFTN2>	-0.55	$1.49*10^{-3}$
nuoH	B<oFabx>	-1.69	$5.10*10^{-8}$
	B<oBIBH1>	-1.07	$1.04*10^{-8}$
	B<oBIWA4>	-0.59	$1.72*10^{-3}$
	B<oFTN2>	-0.59	$1.35*10^{-3}$
nuoI	B<oFabx>	-1.53	$3.44*10^{-7}$
	B<oBIBH1>	-0.95	$1.29*10^{-5}$
	B<oBIWA4>	-0.59	$5.48*10^{-4}$
	B<oFTN2>	-0.65	$2.32*10^{-4}$
nuoJ	B<oFabx>	-1.65	$2.46*10^{-8}$
	B<oBIBH1>	-1.04	$6.00*10^{-8}$
	B<oBIWA4>	-0.64	$3.34*10^{-4}$
	B<oFTN2>	-0.59	$1.26*10^{-3}$
nuoK	B<oFabx>	-1.50	$7.46*10^{-7}$
	B<oBIBH1>	-0.87	$1.72*10^{-5}$
	B<oBIWA4>	-0.46	$1.17*10^{-2}$
	B<oFTN2>	-0.51	$6.52*10^{-3}$

Sophie Vazulka[1], Matteo Schiavinato[2], Martin Wagenknecht[3], Monika Cserjan-Puschmann[1]*, Gerald Striedner[1]

1 Christian Doppler Laboratory for production of next-level biopharmaceuticals in *E. coli*, University of Natural Resources and Life Sciences, Vienna, Department of Biotechnology, Institute of Bioprocess Science and Engineering, Muthgasse 18, 1190 Vienna, Austria

2 University of Natural Resources and Life Sciences, Vienna, Department of Biotechnology, Institute of Computational Biology, Muthgasse 18, 1190 Vienna, Austria

3 Boehringer Ingelheim RCV GmbH & Co KG, Dr.-Boehringer-Gasse 5-11, 1120 Vienna, Austria

nuoL	B<oFabx>	-1.50	$5.20*10^{-7}$
	B<oBIBH1>	-0.99	$4.54*10^{-7}$
	B<oBIWA4>	-0.58	$1.07*10^{-3}$
	B<oFTN2>	-0.56	$1.78*10^{-3}$
nuoM	B<oFabx>	-1.27	$5.85*10^{-6}$
	B<oBIBH1>	-0.79	$4.05*10^{-5}$
	B<oFTN2>	-0.41	$4.78*10^{-3}$
nuoN	B<oFabx>	-0.83	$6.19*10^{-4}$
	B<oBIBH1>	-0.67	$3.03*10^{-4}$
ndh	B<oFabx>	1.73	$9.46*10^{-12}$
	B<oBIWA4>	0.56	$5.52*10^{-4}$

Table S2: GO terms enriched (level 5) in B<oFabx> after 12 h of production in fed-batch processes.

Enriched GO term		Q value	Gene count
Cellular component	GO:0022626 cytosolic_ribosome	$1.85*10^{-20}$	55
	GO:0044391 ribosomal_subunit	$1.85*10^{-20}$	55
	GO:0043232 intracellular_non-membrane-bounded_organelle	$2.87*10^{-18}$	67
	GO:0044445 cytosolic_part	$2.54*10^{-13}$	60
	GO:0030964 NADH_dehydrogenase_complex	$1.16*10^{-4}$	14
	GO:0070470 plasma_membrane_respiratory_chain	$1.14*10^{-4}$	18
	GO:0098803 respiratory_chain_complex	$1.14*10^{-4}$	18
	GO:0045272 plasma_membrane_respiratory_chain_complex_I	$2.08*10^{-4}$	13
	GO:0098797 plasma_membrane_protein_complex	$2.34*10^{-2}$	64
Molecular function	GO:0003723 RNA_binding	$4.33*10^{-6}$	60
	GO:0003954 NADH_dehydrogenase_activity	$2.02*10^{-3}$	16
Biological process	GO:0022618 ribonucleoprotein_complex_assembly	$3.68*10^{-13}$	47
	GO:0071826 ribonucleoprotein_complex_subunit_organization	$3.68*10^{-13}$	48
	GO:0070925 organelle_assembly	$7.75*10^{-13}$	52
	GO:0008652 cellular_amino_acid_biosynthetic_process	$3.65*10^{-5}$	65
	GO:1901605 alpha-amino_acid_metabolic_process	$9.65*10^{-4}$	83
	GO:0009408 response_to_heat	$6.30*10^{-3}$	36
	GO:0044275 cellular_carbohydrate_catabolic_process	$2.73*10^{-2}$	27
	GO:0097237 cellular_response_to_toxic_substance	$3.70*10^{-2}$	12

Table S3: GO terms enriched (level 5) in B<oBIBH1> after 12 h of production in fed-batch processes.

Enriched GO term		Q value	Gene count
Cellular component	GO:0022626 cytosolic_ribosome	$9.01*10^{-14}$	44
	GO:0044391 ribosomal_subunit	$9.01*10^{-14}$	44
	GO:0044446 intracellular_organelle_part	$8.69*10^{-12}$	46
	GO:0044445 cytosolic_part	$2.23*10^{-10}$	48
	GO:0070470 plasma_membrane_respiratory_chain	$1.19*10^{-6}$	18

Sophie Vazulka[1], Matteo Schiavinato[2], Martin Wagenknecht[3], Monika Cserjan-Puschmann[1]*, Gerald Striedner[1]

1 Christian Doppler Laboratory for production of next-level biopharmaceuticals in *E. coli*, University of Natural Resources and Life Sciences, Vienna, Department of Biotechnology, Institute of Bioprocess Science and Engineering, Muthgasse 18, 1190 Vienna, Austria

2 University of Natural Resources and Life Sciences, Vienna, Department of Biotechnology, Institute of Computational Biology, Muthgasse 18, 1190 Vienna, Austria

3 Boehringer Ingelheim RCV GmbH & Co KG, Dr.-Boehringer-Gasse 5-11, 1120 Vienna, Austria

	GO:0098803 respiratory_chain_complex	$1.19*10^{-6}$	18
	GO:0045271 respiratory_chain_complex_I	$5.33*10^{-6}$	13
	GO:0045272 plasma_membrane_respiratory_chain_complex_I	$5.33*10^{-6}$	13
	GO:0098797 plasma_membrane_protein_complex	$1.17*10^{-2}$	51
	GO:0030288 outer_membrane-bounded_periplasmic_space	$2.02*10^{-2}$	54
Molecular function	GO:0003954 NADH_dehydrogenase_activity	$5.42*10^{-4}$	15
Biological process	GO:0070925 organelle_assembly	$3.27*10^{-7}$	40
	GO:0071826 ribonucleoprotein_complex_subunit_organization	$7.73*10^{-6}$	34
	GO:0042273 ribosomal_large_subunit_biogenesis	$1.42*10^{-4}$	20
	GO:0034599 cellular_response_to_oxidative_stress	$2.85*10^{-3}$	18
	GO:0097237 cellular_response_to_toxic_substance	$2.58*10^{-2}$	11

Table S4: GO terms enriched (level 5) in B<oBIWA4> after 12 h of production in fed-batch processes.

Enriched GO term		Q value	Gene count
Cellular component	GO:0022626 cytosolic_ribosome	$2.76*10^{-19}$	55
	GO:0044391 ribosomal_subunit	$2.76*10^{-19}$	55
	GO:0043232 intracellular_non-membrane-bounded_organelle	$6.15*10^{-17}$	67
	GO:0044445 cytosolic_part	$9.02*10^{-14}$	62
	GO:0070470 plasma_membrane_respiratory_chain	$1.96*10^{-2}$	15
	GO:0098803 respiratory_chain_complex	$1.96*10^{-2}$	15
	GO:0098797 plasma_membrane_protein_complex	$2.35*10^{-2}$	67
	GO:0030964 NADH_dehydrogenase_complex	$2.59*10^{-2}$	11
Biological process	GO:0022618 ribonucleoprotein_complex_assembly	$3.78*10^{-11}$	46
	GO:0071826 ribonucleoprotein_complex_subunit_organization	$3.78*10^{-11}$	47
	GO:0070925 organelle_assembly	$6.52*10^{-11}$	51
	GO:0008652 cellular_amino_acid_biosynthetic_process	$1.46*10^{-6}$	71
	GO:1901605 alpha-amino_acid_metabolic_process	$2.88*10^{-4}$	88
	GO:0044275 cellular_carbohydrate_catabolic_process	$8.23*10^{-3}$	29
	GO:0009408 response_to_heat	$1.23*10^{-2}$	36
	GO:0097237 cellular_response_to_toxic_substance	$1.23*10^{-2}$	13
	GO:0010038 response_to_metal_ion	$2.04*10^{-2}$	29

Table S5: GO terms enriched (level 5) in B<oFTN2> after 12 h of production in fed-batch processes.

Enriched GO term		Q value	Gene count
Cellular component	GO:0022626 cytosolic_ribosome	$4.54*10^{-26}$	56
	GO:0044391 ribosomal_subunit	$4.54*10^{-26}$	56
	GO:0044446 intracellular_organelle_part	$4.45*10^{-21}$	59
	GO:0044445 cytosolic_part	$9.54*10^{-19}$	62
	GO:0045271 respiratory_chain_complex_I	$3.60*10^{-4}$	12
	GO:0045272 plasma_membrane_respiratory_chain_complex_I	$3.60*10^{-4}$	12
	GO:0070470 plasma_membrane_respiratory_chain	$4.03*10^{-4}$	16

Sophie Vazulka[1], Matteo Schiavinato[2], Martin Wagenknecht[3], Monika Cserjan-Puschmann[1]*, Gerald Striedner[1]

1 Christian Doppler Laboratory for production of next-level biopharmaceuticals in *E. coli*, University of Natural Resources and Life Sciences, Vienna, Department of Biotechnology, Institute of Bioprocess Science and Engineering, Muthgasse 18, 1190 Vienna, Austria

2 University of Natural Resources and Life Sciences, Vienna, Department of Biotechnology, Institute of Computational Biology, Muthgasse 18, 1190 Vienna, Austria

3 Boehringer Ingelheim RCV GmbH & Co KG, Dr.-Boehringer-Gasse 5-11, 1120 Vienna, Austria

	GO:0098803 respiratory_chain_complex	$4.03*10^{-4}$	16
Biological process	GO:0070925 organelle_assembly	$7.11*10^{-15}$	51
	GO:0071826 ribonucleoprotein_complex_subunit_organization	$3.64*10^{-13}$	45
	GO:0042273 ribosomal_large_subunit_biogenesis	$2.48*10^{-7}$	24
	GO:1901605 alpha-amino_acid_metabolic_process	$1.31*10^{-3}$	73
	GO:0097237 cellular_response_to_toxic_substance	$1.04*10^{-2}$	12
	GO:0010038 response_to_metal_ion	$1.26*10^{-2}$	26
	GO:0034599 cellular_response_to_oxidative_stress	$2.37*10^{-2}$	17
	GO:0044275 cellular_carbohydrate_catabolic_process	$3.08*10^{-2}$	24
	GO:0009308 amine_metabolic_process	$3.08*10^{-2}$	18
	GO:0009408 response_to_heat	$3.08*10^{-2}$	30

Table S6: Primers used for qPCR.

Target	Sequence of forward primer	Sequence of reverse primer	Amplicon length [bp]
soxS	5‘-CAAGCGTCTGATGCGTCACC-3‘	5‘-CGAGCATATTGACCAGCCGC-3‘	107
cysG	5‘-GGTTGCTGTTAGACGCAGGC-3‘	5‘-CATCTGCCCATGCGGTGAAC-3‘	78
